# Application of General Unified Threshold Models of Survival Models for Regulatory Aquatic Pesticide Risk Assessment Illustrated with an Example for the Insecticide Chlorpyrifos

**DOI:** 10.1002/ieam.4327

**Published:** 2020-09-24

**Authors:** Theo Brock, Maria Arena, Nina Cedergreen, Sandrine Charles, Sabine Duquesne, Alessio Ippolito, Michael Klein, Melissa Reed, Ivana Teodorovic, Paul J van den Brink, Andreas Focks

**Affiliations:** ^1^ Wageningen Environmental Research Wageningen the Netherlands; ^2^ European Food Safety Authority Parma Italy; ^3^ University of Copenhagen Copenhagen Denmark; ^4^ University of Lyon Lyon France; ^5^ Umweltbundesamt Dessau Germany; ^6^ Fraunhofer IME Schmallenberg Germany; ^7^ Chemicals Regulation Division‐HSE York United Kingdom; ^8^ University of Novi Sad Novi Sad Serbia; ^9^ Wageningen University Wageningen the Netherlands

**Keywords:** Toxicokinetic–toxicodynamic modeling, GUTS, Chlorpyrifos, Pesticide risk assessment, Time‐variable exposure

## Abstract

Mathematical models within the General Unified Threshold models of Survival (GUTS) framework translate time‐variable chemical exposure information into expected survival of animals. The GUTS models are species and compound specific and explicitly describe the internal exposure dynamics in an organism (toxicokinetics) and the related damage and effect dynamics (toxicodynamics), thereby connecting the external exposure concentration dynamics with the simulated mortality or immobility over time. In a recent scientific opinion on toxicokinetic–toxicodynamic (TKTD) models published by the European Food Safety Authority (EFSA), the GUTS modeling framework was considered ready for use in the aquatic risk assessment for pesticides and aquatic fauna. The GUTS models are suggested for use in risk assessment, if they are sufficiently validated for a specific substance–species combination. This paper aims to illustrate how they can be used in the regulatory environmental risk assessment for pesticides for a specific type of refinement, that is, when risks are triggered by lower tiers in acute as well as in chronic risk assessment and mortality or immobility is the critical endpoint. This approach involves the evaluation of time‐variable exposure regimes in a so‐called “Tier‐2C” assessment. The insecticide chlorpyrifos was selected as an example compound because a large data set was available. The GUTS models for 13 different freshwater arthropods and 8 different theoretical aquatic exposure profiles were used to calculate a series of GUTS‐based risk estimates, including exposure profile‐specific multiplication factors leading to 50% mortality or immobility at the end of the tested profile (LP50/EP50) as “margins of safety.” To put the use of GUTS models within the tiered aquatic risk assessment into perspective, GUTS models for the 13 aquatic arthropods were also used to predict the environmental risks of a measured chlorpyrifos exposure profile from an experimental ditch study (Tier‐3 approach), and the results are discussed in the context of calibration of the tiered approach. *Integr Environ Assess Manag* 2021;17:243–258. © 2020 The Authors. *Integrated Environmental Assessment and Management* published by Wiley Periodicals LLC on behalf of Society of Environmental Toxicology & Chemistry (SETAC)

## KEY POINTS

- This paper illustrates how General Unified Threshold models of Survival (GUTS) models can be used in the regulatory environmental risk assessment for pesticides for the evaluation of time‐variable exposure regimes in a so‐called Tier‐2C assessment.
- For chlorpyrifos as an example compound, GUTS models for 13 different freshwater arthropods and 8 different theoretical aquatic exposure profiles were used to calculate a series of GUTS‐based risk estimates, including exposure profile‐specific “margins of safety” (LP50/EP50) values.
- GUTS models were put in perspective within the tiered aquatic risk assessment by comparing the predicted environmental risks of a measured chlorpyrifos exposure profile for 13 aquatic arthropods to results from an experimental ditch study.
- For the example data set (chlorpyrifos) the results of the Tier‐2C assessment using GUTS models were not in conflict with the principle of the tiered approach in that lower tiers should be more conservative than higher tiers.

## INTRODUCTION

Plant protection products (PPPs) are subject to prospective risk assessment procedures before they are authorized for the European market. Environmental risk assessment (ERA) aims at identifying possible significant negative impacts of PPPs on the environment and is usually performed for a number of compartments and specific biological groups, including aquatic organisms, nontarget arthropods and terrestrial plants, soil organisms, birds and mammals, and bees. The ERA procedures for the aquatic environment in Europe follow a tiered approach, where initial lower tier methods give risk estimates that require less time and economic investment; these estimates are supposed to be conservative. When a potentially high risk of a PPP for an intended use is indicated in a lower tier, higher tier procedures are proposed as a refinement option (EFSA PPR 2013).

Chemical risk assessment commonly separates the exposure from the effect analysis. While exposure analysis has used mechanistic mathematical models for decades, effect assessment was predominately based on experiments and simple, purely descriptive statistical models. In 2018, the Panel on Plant Protection Products and their Residues (PPR) of the European Food Safety Authority (EFSA) concluded that, based on the current state of the art, the mechanistic General Unified Threshold models of Survival (GUTS) modeling framework (Jager et al. 2011; Jager and Ashauer 2018) is ready to be used in the aquatic risk assessment for pesticides and aquatic fauna (invertebrates, fish, aquatic stages of amphibians). Indeed, GUTS models have been found to be a solid method for the evaluation of effects of time‐variable chemical exposure on the survival of aquatic organisms, also addressing uncertainties in the calculation of ERA endpoints in a more elaborated way than in experiment‐based methods (Ashauer et al. 2016; EFSA PPR et al. 2018; Baudrot and Charles 2019). Furthermore, GUTS models for substance–species combinations of interest can be used as a refinement option in risk assessment if they are sufficiently validated. According to EFSA PPR et al. (2018), GUTS models are fit‐for‐purpose to be applied in acute or chronic risk assessment for PPPs and aquatic fauna, under the condition that mortality or immobility is the critical (i.e., most sensitive) endpoint, if risks are triggered by lower tiers. The GUTS models cannot be used for the risk assessment of PPPs and aquatic primary producers. For this, species‐specific macrophyte models such as the *Lemna* model by Schmitt et al. (2013) could be applied, given that more validation examples become available, optimization methods would be described in more detail, and sensitivity analyses become available to allow for a comprehensive understanding of the variation in the model parameters (EFSA PPR et al. 2018). For sublethal endpoints and the aquatic fauna, DEBtox models are under observation as a refinement option (EFSA PPR et al. 2018). The GUTS models, in addition to the specific application in risk assessment, also can be used also to address other regulatory aspects, for example, to evaluate the possible toxicological (in)dependence of different exposure pulses.

The GUTS models are species and compound specific and follow the principle that the processes governing the internal exposure within an organism, summarized by the term “toxicokinetics” (TK), can be described separately from the processes that lead to damage and effects, summarized by the term “toxicodynamics” (TD). The ultimate aim of GUTS models is to predict the survival probability of exposed individuals for the species of concern, under untested exposure conditions. Originally, GUTS models were developed for assessing the effects of time‐variable exposure time series, but in addition the potential of GUTS for an improved Tier‐1 assessment or for extrapolation of Tier‐1 short‐term tests to longer durations under constant exposure conditions is increasingly recognized. Based on the GUTS modeling framework, external concentrations of a chemical are translated into simulated mortality or immobility. Within the full GUTS model, TK processes are described according to the internal concentrations, which are then connected to the so‐called “damage dynamics.” Because internal concentrations, however, are rarely measured in standard toxicity tests conducted for risk assessment purposes, and hence notoriously difficult to be used for model calibration, reduced GUTS versions (GUTS‐RED) were developed, in which external concentrations are directly connected to the damage dynamics. Two reduced versions of GUTS are available: the GUTS‐RED‐SD version, based on the assumption of stochastic death (SD), and the GUTS‐RED‐IT version, based on the assumption of individual tolerance (IT). In GUTS‐RED‐SD, the threshold parameter for lethal effects is fixed and identical for all individuals, meaning that the variance of the threshold values for mortality or immobility is zero, but the probability of dying linearly increases for each individual according to the increasing internal damage. In contrast, in GUTS‐RED‐IT, threshold values for effects are distributed among individuals and once an individual tolerance is exceeded, mortality or immobility of this individual follows immediately. For a more detailed description of the GUTS modeling framework, refer to Ashauer et al. (2016), Jager and Ashauer (2018), and EFSA PPR et al. (2018). An advantage of the GUTS‐RED model versions is that their calibration can be conducted by using the exposure‐response dynamics from standardized laboratory single‐species tests (Ashauer et al. 2013; Baudrot, Preux et al. 2018; Baudrot, Veber et al. 2018; Focks et al. 2018; Jager and Ashauer 2018).

The present paper aims to illustrate how reduced GUTS models can be used to refine the regulatory environmental risk assessment for pesticides in case of time‐variable exposure regimes. This appears relevant because short‐term pulse exposures to pesticides in edge‐of‐field surface waters are more often the rule than the exception (see, e.g., Brock et al. 2010), whereas lower tier standard laboratory toxicity tests follow a more conservative approach by using constant exposure concentrations. We selected the insecticide chlorpyrifos (CPF) as the benchmark compound, given that information on concentration–response relationships during 96‐h laboratory toxicity tests exists for a wide array of freshwater arthropods (Rubach et al. 2011) and given that it was used as one of the compounds for the characterization of exposure pattern‐specific species sensitivity distributions (SSDs) (Van den Brink et al. 2019).

In the present paper, we used the CPF data set to illustrate the Tier‐2C risk assessment procedure based on the GUTS modeling framework as proposed by EFSA PPR et al. (2018). In particular, reduced GUTS models for 13 different freshwater arthropods and 8 different theoretical aquatic exposure profiles, all characterized by the same peak concentration but different in the 28‐d overall exposure, were used to illustrate the potential of the GUTS modeling framework to assess the risk of time‐variable exposures. In addition, to put this Tier‐2C risk assessment into perspective of the tiered approach, the calibrated GUTS‐RED models for 13 aquatic arthropods were further used to predict the environmental risks of a measured CPF exposure profile from an experimental ditch study that allowed the derivation of a provisional Tier‐3 regulatory acceptable concentration (RAC) following the procedure described in the aquatic guidance document (AGD) of EFSA (EFSA PPR 2013). In the present paper, references in terms of toxicity and exposure are made to information available from the European Union (EU) review report (EC 2005), which might be partly outdated. As such, the present paper should not be considered as an attempt to address the actual aquatic risk resulting from the use of CPF, but rather it makes use of CPF as a model substance to illustrate the theoretical use of GUTS modeling as the Tier‐2C approach in the risk assessment procedure used when evaluating active substances.

Hence, our paper provides calculation examples related specifically to the implementation of GUTS into the risk assessment; it does not provide examples related to the validation of the application of GUTS for CPF. Background information and corresponding examples for GUTS validation can be found in the recent EFSA scientific opinion on TKTD models (EFSA PPR et al. 2018) or in the open literature (e.g., Focks et al. 2018). In addition, it should be noted that concerns related to the Tier‐2C approach are not addressed in the present paper because its aim is neither to promote the Tier‐2C approach nor to critically assess it, but instead it is to evaluate the possibility of GUTS modeling as a tool in regulatory risk assessment.

## MATERIALS AND METHODS

### Laboratory toxicity data for CPF

Aquatic arthropods are the most sensitive taxonomic group to the active substance CPF. Raw data on lethality or immobility from 96‐h single‐species toxicity tests with CPF and 13 different freshwater arthropods, as published by Rubach et al. (2011), were used in the assessments presented in the present paper (see Table 1). Immobility data were corrected for recovery when necessary. Indeed, because GUTS models cannot handle individual recovery, individuals are considered immobile for the rest of the test period once they have shown immobility. This was the case for some replicates of 3 tested organisms: *Neocaridinia denticulata, Paraponyx stratiotata* and *Procambarus* sp. Quality criteria for the calibration data as laid down in the EFSA TKTD scientific opinion (EFSA PPR et al. 2018) were checked: Maximum effects for all tested species were close to 100%, and survival and mobility were checked at 5 time points (initially and on 4 consecutive days).

**Table 1:** Summary information for the 13 aquatic arthropod taxa from which the concentration–response relationships (endpoint immobility) of 96‐h toxicity tests (data from Rubach et al. 2011) were used to parametrize the GUTS‐RED models^a^

Species	Taxonomic group	96‐h EC50 in µg/L (95% CI)	Used in ERA tiers^b^
*Asellus aquaticus*	Crustacea, Isopoda	3.43 (2.75–4.26)	Tier‐2A; Tier‐2B Tier‐C_2_
*Daphnia magna*	Crustacea, Cladocera	0.17 (0.12–0.23)	Tier‐1; Tier‐2A; Tier‐2B Tier‐C_1_; Tier‐C_2_
*Neocardinia denticulata*	Crustacea, Decapoda	171 (NC)	Tier‐2B Tier‐C_2_
*Gammarus pulex*	Crustacea, Amphipoda	0.23 (0.20–0.25)	Tier‐2A; Tier‐2B Tier‐C_2_
*Procambarus* sp.	Crustacea, Decapoda	1.20 (0.75–1.93)	Tier‐2B Tier‐C_2_
*Anax imperator*	Insecta, Odonata	1.63 (NC)	Tier‐2B Tier‐C_2_
*Cloeon dipterum*	Insecta, Ephemeroptera	0.31 (0.26–0.38)	Tier‐2A; Tier‐2B Tier‐C_2_
*Chaoborus obscuripes* c	Insecta, Diptera	0.18 (0.07–0.43)	Tier‐1; Tier‐2A; Tier‐2B Tier‐C_1_; Tier‐C_2_
*Notonecta maculata*	Insecta, Hemiptera	2.78 (NC)	Tier‐2B Tier‐C_2_
*Paraponyx stratiotata*	Insecta, Lepidoptera	2.86 (1.17–6.97)	Tier‐2B Tier‐C_2_
*Plea minutissima*	Insecta, Hemiptera	1.29 (0.92–1.80)	Tier‐2A; Tier‐2B Tier‐C_2_
*Ranatra linearis*	Insecta, Hemiptera	3.33 (2.95–3.76)	Tier‐2B Tier‐C_2_
*Sialis lutaria*	Insecta, Megaloptera	0.96 (NC)	Tier‐2B Tier‐C_2_

ERA = environmental risk assessment; GUTS‐RED = reduced General Unified Threshold models of Survival; NC = confidence interval could not be calculated.

^a^ The 96‐h EC50 values were also used in different ERA tiers based on experimental data.

^b^ Experimental tiers: Tier‐1 = standard test species approach; Tier‐2A = geometric mean approach; Tier‐2B =species sensitivity distribution approach. Tiers based on GUTS models for standard (Tier‐C_1_) and standard and additional species (Tier‐2C_2_).

^c^ Toxicity value used as a proxy for the Tier‐1 test species *Chironomus riparius*.

### Aquatic exposure profiles

**Figure 1:**
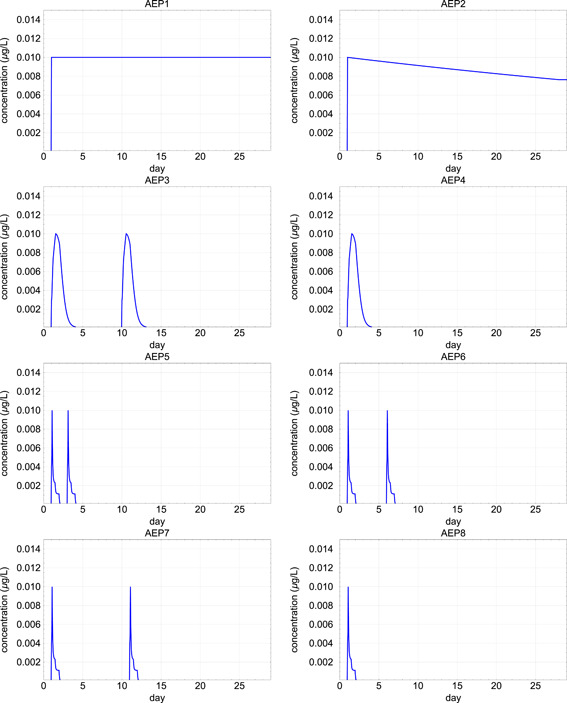
Chlorpyrifos concentrations (in µg/L) over time for 8 theoretical aquatic exposure profiles (AEP1–AEP8). All constructed AEPs have the same peak concentration but are characterized by a decreasing overall exposure when going from AEP1 to AEP8 and/or a larger distance between repeated pulse exposures.

To illustrate how GUTS‐RED models can be used in aquatic risk assessment, but also to evaluate the impact of different types of insecticide exposure patterns that may occur in edge‐of‐field surface waters, we constructed 8 different 28‐d aquatic exposure profiles (AEPs). They were all characterized by the same peak concentration of 0.01 µg/L of CPF, but differed in the frequency and duration of and the time interval between exposure peaks, that is, in the overall exposure (see panels AEP1 to AEP8 in Figure 1). For illustrative purposes, we selected a time frame of 28 d, which is inspired by the duration of chronic toxicity tests for aquatic insects (see, e.g., the chronic Organisation for Economic Co‐operation and Development test with *Chironomus* sp.; OECD 2010) and includes all pulses in AEP3 to AEP8. In edge‐of‐field surface waters, CPF may show relatively fast dissipation in the water compartment, resulting in a single or a few repeated exposure peaks shorter than 28 d (see, e.g., Crum and Brock 1994; López‐Mancisidor et al. 2008). The 8 theoretical aquatic exposure profiles in Figure 1 are arranged in the order from the highest expected risk (AEP1, with the highest overall exposure) to the lowest expected risk (AEP8, with the lowest overall exposure). Panels AEP5, AEP6, and AEP7 are characterized by 2 pulse exposures and the same 28‐d overall exposure, but the time intervals between pulses increase from AEP5 to AEP7. We included AEP1 characterized by a constant exposure regime (and consequently the highest overall exposure) because in standardized laboratory toxicity tests, from which the data used for the GUTS‐RED model calibration initially originate, the exposure concentration is assumed to be constant.

### Model equations and calibration

Model calibration for the 13 arthropod taxa was done using immobility data and concentration measurements of external exposure. Scaled internal damage was used (Eqn. 1) and linked to the stochastic death model (GUTS‐RED‐SD; Eqns. 2 and 3) or to the individual tolerance model (GUTS‐RED‐IT; Eqns. 4 and 5) to describe the survival frequencies over time (see Jager et al. 2011).

(1) $$\frac{{\mathrm{dD}}_{w}(t)}{\mathrm{dt}}=k_{D}{\;}_{*}\;(C_{\mathrm{ext}}\left((,t,)\right)-D_{w}\left(t\right)).$$

In Equation 1, *D*_*w*_ *(t)* is the scaled internal concentration in concentration units, *C*_*ext*_(*t*) the external concentration in the water (concentration units), and *k*_*D*_ the dominant rate constant (per time unit).

(2) $$\frac{\mathrm{dH}(t)}{\mathrm{dt}}=b_{w}{\;}_{*}\;\max(0,D_{w}\left(t\right)-z_{w}),$$

(3) $$S_{\mathrm{SD}}\left(t\right)=e^{-H(t)}{\;}_{*}\;e^{-h_{b}\;{\;}_{*}t}.$$

In Equations 2 and 3, *S*(*t*) is the survival probability (between 0 and 1, dimensionless but usually expressed in %); *H*(*t*) the cumulated hazard rate at time *t* (per time unit); *b*_*w*_ the killing rate constant (per concentration and time unit); *z*_*w*_ the threshold (concentration unit); and *h*_*b*_ the background mortality rate constant (per time unit).

(4) $$F\left(t\right)=\frac{1}{1+{\left(\frac{\max_{0\leq \tau \leq t\;}D_{w}\left(\tau \right)}{m_{w}}\right)}^{-\beta }},$$

(5) $$S_{\mathrm{IT}}\left(t\right)=\left((,1-F\left(t\right),)\right){\;}_{*}\;e^{-h_{b}\;{\;}_{*}t}.$$

In Equations 4 and 5, *F*(*t*) is the cumulative distribution function at time *t, h*_*b*_ the background mortality rate constant (per time unit), *k*_*D*_ the dominant rate constant (per time unit), *m*_*w*_ the median threshold (concentration unit), and *β* the spread of the threshold distribution (dimensionless).

For the optimization of the parameters, the likelihood function derived by Jager et al. (2011) was used (Eqn. 6):

(6) $$\ln\;l\left(y|\Theta \right)=\sum_{i=1}^{n+1}\left(y_{i-1}-y_{i})\times \ln\;(S_{i-1}\left(t,\Theta \right)-S_{i}\left(t,\Theta \right)\right).$$

In Equation 6, *y*_*i*_ is the experimental observation at time point *i, S*(*t, Θ*) the theoretical survival probability, and *Θ* the parameter vector (or parameter set): *Θ* = (*k*_*D*_, *b, z*_*w*_, *h*_*b*_) for GUTS‐RED‐SD and *Θ* = (*k*_*D*_, *m*_*w*_, *β, h*_*b*_) for GUTS‐RED‐IT.

The optimal vector *Θ*_*opt*_ for each of the species‐specific GUTS‐RED‐SD and GUTS‐RED‐IT models corresponds to parameter values that match the experimentally observed survival data. For convenience, background mortality rate constants were here fitted to survival data in controls separately. Fitting the background mortality rate constants to survival in the controls assumes that only control data may provide information on this parameter, while this approach ignores possible correlations between the background mortality rate constant and the 3 other parameters. So, alternatively, *h*_*b*_ can be fitted simultaneously with the other parameters (Baudrot and Charles 2019). In the present paper, when no mortality at all occurred during the toxicity tests, the background mortality probability was fixed to 0.005/d which corresponds to an average maximum arbitrary lifespan of 200 d. Next, survival probabilities were calculated in explicit dependence of the parameter vectors *Θ* (see above Equation 6; excluding *h*_*b*_ when useful) as well as of the measured external concentration over time. Survival in small cohorts follows a multinomial distribution (or equivalently a conditional binomial distribution), and the log‐likelihood function was applied for measuring the agreement between model and observations. In the present paper, optimization was done using the built‐in optimization routine Simulated Annealing of the method NMinimize of Mathematica (Wolfram Research, version 11.3; see https://reference.wolfram.com/language/ref/NMinimize.html) using the options PerturbationScale = 3, SearchPoints = 25, PrecisionGoal = 6, MaxIterations = 10. Parameter sets were obtained for the best fits between data and model simulations by minimization of the negative log‐likelihood function (Eqn. 6). The likelihood ratio method was then used to numerically approximate confidence intervals for the optimal parameter *Θ*_*opt*_ (Meeker and Escobar 1995; Albert et al. 2012; Ashauer et al. 2016).

### Calculation of exposure profile‐specific multiplication factors

Following the proposal by EFSA PPR et al. (2018), we calculated exposure profile‐specific multiplication factors leading to 50% mortality or immobility at the end of the tested profile (namely LP50/EP50) for all theoretical exposure profiles presented in Figure 1. The LP50/EP50 concept is based on the margins of safety concept as presented in Ashauer et al. (2013). Analogous to the LC50/EC50 calculation of a laboratory test with more or less constant exposure, which reports the midpoint of the dose–response relationship and so the concentration that causes 50% mortality or immobility in comparison to the control, the LP50/EP50 value quantifies the effect of a certain entire time‐variable exposure profile integrated over time and leading to 50% mortality or immobility at the end of the exposure. Attention is needed because LC50/EC50 values are concentrations, while LP50/EP50 values refer to multiplication factors applied to specific time‐variable exposure profiles. Varying the multiplication factors, the whole exposure profile can be shifted in height until it reaches the factor that will result in exactly 50% mortality or immobility at the end of the exposure duration. The LP50/EP50 values for the 8 theoretical aquatic exposure profiles (Figure 1) were calculated using both the GUTS‐RED‐SD and GUTS‐RED‐IT models.

### Data analysis and ERA approach

In order to put the aquatic risk assessments based on GUTS‐RED models into regulatory perspective, we derived provisional Tier‐1 (standard test species approach), Tier‐2A (geometric mean approach, also geomean approach), Tier‐2B (SSD approach) and Tier‐3 (model ecosystem approach) RACs in line with the guidance provided in EFSA PPR (2013). This was done here for illustrative purpose alone, because in practice not all tiers may be needed; for example, if enough data are available for conducting an SSD, the geomean is not used. For use in risk assessment, GUTS models need to be validated against independent measurements in laboratory experiments, which is not done in the present manuscript. Further consideration on this point is included in the *Discussion* section.

In the current guidance for aquatic risk assessment (EFSA PPR 2013), RAC values are in general derived by selecting a predefined toxicity estimate and dividing it by an appropriate assessment factor (AF). The RAC is then compared to the maximum predicted exposure concentration (PEC_max)_ value; if the RAC is higher than the PEC_max_, low risk is indicated. This procedure is numerically identical to the calculation of toxicity exposure ratio (TER) values that are compared to the same AF. In the present paper, we focused on the calculation of TERs, because they provide values comparable to the profile‐specific LP50/EP50 values that results from the GUTS modeling framework.

**Figure 2:**
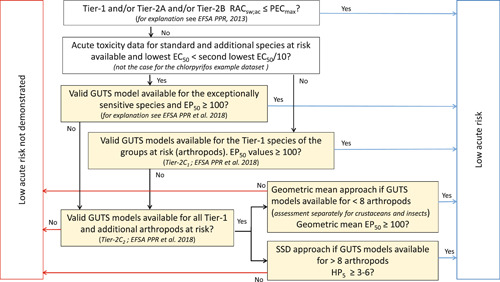
Decision scheme for the use of GUTS‐RED models and estimated EP50 and HP5 values in the tiered acute ERA for the example insecticide chlorpyrifos (adapted after EFSA PPR et al. 2018). The steps based on GUTS models (Tier‐2C_1_ and Tier‐2C_2_) are shaded. AEP = aquatic exposure profile; EFSA = European Food Safety Authority; GUTS‐RED = reduced General Unified Threshold models of Survival; PEC_max_ = highest predicted exposure concentration; RAC_sw;ac_ = regulatory acceptable concentration in surface water and the acute effects assessment.

We calculated TERs based on the experimental acute toxicity data presented in Table 1 and the aquatic exposure profiles presented in Figure 1. In these TERs, the toxicity estimate can be 1) the 96‐h EC50 of the most sensitive standard test species in Tier‐1, 2) the lowest geometric mean 96‐h EC50 for either crustaceans or insects in Tier‐2A, or 3) the median hazardous concentration for 5% of the tested species (HC5) in Tier‐2B. The exposure estimate of the TER is always the PEC_max_. For consistency, 96‐h EC50 values (endpoint immobility) were used in all experimental effect assessment tiers based on laboratory toxicity tests because the data underlying these 96‐h EC50 values were also used to calibrate the GUTS‐RED models. In the acute risk assessments on basis of the available GUTS‐RED models (Tier‐2C), we used the decision scheme in line with EFSA PPR et al. (2018) (see Figure 2).

In the acute toxicity data set for the insecticide CPF, the standard test species of arthropods are an order of magnitude more sensitive than that of the noninvertebrate standard test species (e.g., fish and algae) (EC 2005; Giddings et al. 2014). In the acute Tier‐1 effect assessment, toxicity data for at least 2 aquatic arthropod taxa are required for a substance with an insecticidal mode of action, viz., for the cladoceran *Daphnia* sp. and another species (see Regulation 283/2013 data requirement [EC 2013] and EFSA PPR 2013); the other species can be standard test species, for example, *Chironomus* sp. or *Americamysis bahia*, or any other species being highly sensitive compared to the standard test species found in the open literature. In this example case with CPF, the most sensitive crustacean in literature overlapped with the standard species (*Daphnia*), while for the selection of the most sensitive insect, the acute LC50/EC50 values reported for *Chironomus* sp. and CPF in the open literature (Giddings et al. 2014), resemble very much the acute toxicity values reported by Rubach et al. (2011) for the phantom midge *Chaoborus obscuripes*. It would be preferable to use *Chironomus* sp. here, but the raw data of the concentration–response curves over time could not be made available after consulting the laboratories where these tests were performed, hence GUTS models could not be calibrated for *Chironomus* sp. Because acute LC50/EC50 values for *C. obscuripes* are of comparable sensitivity to those of *Chironomus* sp., and because raw toxicity data over time were available for *C. obscuripes*, we used the 96‐h EC50 value of and the underlying concentration–response data from *C. obscuripes* in our provisional Tier‐1 (experimental standard test species approach) and Tier‐2C_1_ approaches (using GUTS models for the standard test species of the sensitive taxonomic group).

The Tier‐1 TER is the lowest 96‐h EC50 for the combination *Daphnia magna* and *Chaoborus obscuripes* divided by the PEC_max_. This Tier‐1 TER should be larger than 100—the AF in the Tier‐1 RAC derivation—to consider the acute risks as low. In analogy to this, using the GUTS‐RED models for these 2 standard test species of the most sensitive taxonomic group, the Tier‐2C_1_ assessment is based on the calculation of exposure profile‐specific EP50 values with GUTS‐RED models for *D. magna* and *Chaoborus obscuripes*. These EP50 values should be equal to or larger than the acute Tier‐1 AF of 100 to consider the acute risk of the evaluated exposure profile low (see decision scheme in Figure 2).

To illustrate the Tier‐2A approach (experimental geometric mean approach if toxicity data are available for fewer than 8 taxa of the sensitive taxonomic group) and the Tier‐2C_2_ geomean approach (based on GUTS‐RED models for <8 taxa of the sensitive taxonomic group), 96 h‐EC50 values for 3 crustaceans and 3 insects were selected from the Rubach et al. (2011) database for illustrative purposes, whereas in a realistic regulatory risk assessment the geomean would not be the chosen option if sufficient species (>8) for an SSD are available. The selected crustaceans comprised *D. magna, Asellus aquaticus*, and *Gammarus pulex*, whereas the insects comprised *Chaoborus obscuripes, Cloeon dipterum*, and *Plea minutissima* (these taxa are relatively frequently tested in insecticide studies; Van Wijngaarden et al. 2015). Following the procedure proposed by EFSA PPR (2013), the geometric mean 96‐h EC50 values were calculated separately for crustaceans and insects, and the lowest value (either for crustaceans or insects) was selected for Tier‐2A TER derivation. The Tier‐2A TER is thus the lowest geometric mean 96‐h EC50 value divided by the PEC_max_. If this Tier‐2A TER is larger than 100 (the Tier‐1 AF), acute risk is considered low. In analogy to this, using the GUTS‐RED models for these 3 crustaceans and 3 insects in the Tier‐2C_2_ geomean approach requires the calculation of exposure profile‐specific EP50 values for these taxa. The exposure profile‐specific geometric mean EP50 values for both taxonomic groups separately (crustaceans and insects) should be greater than or equal to 100 (the Tier‐1 AF) to consider the acute risk of the evaluated exposure profile low (see decision scheme in Figure 2).

In the acute Tier‐2B (SSD) approach for insecticides, valid EC50 values for at least 8 arthropod taxa should be available (EFSA PPR 2013). With 96‐h EC50 values for 13 different arthropods in the CPF data set (Table 1), this criterion is met. The HC5 (hazardous concentration to 5% of the tested species), derived from the SSD constructed with 96‐h EC50 values for these 13 arthropods, is selected as the toxicity estimate for the Tier‐2B TER derivation, the exposure estimate being again the PEC_max_. If this Tier‐2B TER is larger than 3 to 6, which is the range in AF in the acute effect assessment on basis of the SSD approach for invertebrates according to EFSA PPR (2013), the acute risks are considered low. Analogous to this, the GUTS‐RED models for all 13 aquatic arthropods were used to calculate exposure profile‐specific EP50 values for each species Tier‐2C_2_ SSD approach. These EP50 values were used as input data to construct exposure profile‐specific SSDs and to calculate exposure profile‐specific HP5 values, that is, the hazardous profile for 5% of the tested species expressed in terms of an exposure profile‐specific multiplication factor that is needed to reach this effect at the end of the tested profile. In case of acute ERA and aquatic invertebrates, the exposure profile‐specific median HP5 value should be equal to or larger than the AF used in the Tier‐2B effect assessment, namely 3 to 6 (see decision scheme in Figure 2). For constructing SSDs and calculating corresponding HC5 and HP5 values, we used the MOSAIC Web interface for statistical analysis in ecotoxicology (Charles et al. 2018). The statistical module that MOSAIC offers for SSDs (MOSAIC_SSD_) enables the selection of a log‐normal and a log‐logistic model. We always selected the log‐normal model. The MOSAIC_SSD_ is able to calculate HC5 or HP5 values even if the input data are censored, that is, entered as intervals. In using MOSAIC_SSD_, we thus constructed the SSDs with the lower and upper bounds of the 95% confidence intervals of the EC50 or EP50 estimates for each species. If for a specific species the 95% confidence interval of the 96‐h EC50 was not provided in the data set of Rubach et al. 2011 (see Table 1), the point estimates were used instead for both the lower and upper bounds of the confidence interval.

**Figure 3:**
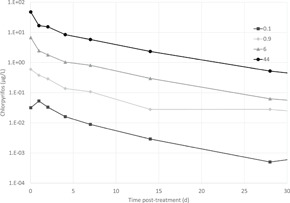
Dynamics of mean concentrations of chlorpyrifos in depth‐integrated water samples in experimental ditches treated with 0.1, 0.9, 6, and 44 µg a.s./L (adapted from Van Wijngaarden et al. 1996). The 0.1 µg a.s./L exposure profile is used to evaluate the conservativeness of the GUTS models. GUTS = General Unified Threshold models of Survival.

A Tier‐3 experimental ditch study on the effects of a single application of CPF on the invertebrate community and on invertebrate populations (Van den Brink et al. 1996; Van Wijngaarden et al. 1996) was used to put the Tier‐2C risk assessment based on GUTS models into perspective. According to the principles of the tiered approach, a Tier‐2C risk assessment should not be less conservative than a Tier‐3 risk assessment (see also EFSA PPR et al. 2018). This can be evaluated by using the exposure profile of the experimental ditch study from which the no observed effect concentration (NOEC) endpoint used in the risk assessment for addressing the threshold option is derived. In the experimental ditch study, CPF was applied once and 4 exposure concentrations were studied (treatment levels of 0.1, 0.9, 6, and 44 µg a.s./L). Dynamics of mean measured concentrations of CPF in the experimental ditches treated with different concentrations are presented in Figure 3. The experimental ditch study had a diverse community of aquatic invertebrates (for details, see Van den Brink et al. 1996). According to EFSA PPR (2013), to derive an effect threshold, Effect class 1 and Effect class 2 concentrations can be used by applying an AF of 2 or 3. In the experimental ditch study, by using principle response curves (Van den Brink and Ter Braak 1999) an NOEC (Effect class 1) of 0.1 µg/L (in terms of peak concentration of the active substance [a.s.]) could be derived for both the zooplankton and macroinvertebrate communities. At the population level, the most sensitive aquatic arthropod (*G. pulex*) showed a small treatment‐related effect on an isolated sampling (Effect class 2) in the 0.1 µg a.s./L treatment, while for all other invertebrate taxa a treatment‐related effect could not be demonstrated. At higher treatment levels (0.9 µg a.s./L and higher), effects on the *G. pulex* population were long term, while several other arthropods (e.g., *Daphnia galeata, Cloeon dipterum, Caenis horaria*) also showed pronounced treatment‐related effects on consecutive samplings (for details, see Van den Brink et al. 1996). Consequently, the exposure profile with a peak concentration of 0.1 µg a.s./L from the experimental ditch study can be used to derive a provisional Tier‐3 regulatory acceptable concentration in accordance with the ecological threshold option (ETO‐RAC) and to evaluate the conservativeness of the Tier‐2C risk assessment based on the available GUTS‐RED models for aquatic arthropods.

Because some of the arthropods reported in the laboratory toxicity studies (Rubach et al. 2011) were also present in the mesocosm results, the consistency of the overall GUTS modeling approach was further tested. Measured exposure profiles in the mesocosm were used as input for the reduced GUTS models for 4 species: *Cloeon dipterum, Chaoborus obscuripes, G. pulex*, and *Asellus aquaticus*. The predicted survival was checked against the results of the mesocosm in terms of per cent abundance versus the control after 4 wk (Van den Brink et al. 1996; Van Wijngaarden et al. 1996).

## RESULTS

### Model calibration

Model calibration to raw data on immobility of 13 aquatic invertebrate species over time resulted in a set of parameters for the GUTS‐RED‐SD and the GUTS‐RED‐IT models including confidence limits (Supplemental Data SI Table 1), and visual inspection of the fitted data (Supplemental Data Figures SI‐1 and SI‐2) indicated plausible fits. Neither the GUTS‐RED‐SD nor the GUTS‐RED–IT model fitted the data better than the other because for 6 of the 13 tested species of the GUTS RED‐SD model and for the remaining 7 species of the GUTS‐RED‐IT model, fitting resulted in lower log‐likelihood values (Supplemental Data SI Table 1).

### Relationship between exposure profile pattern and GUTS output

**Figure 4:**
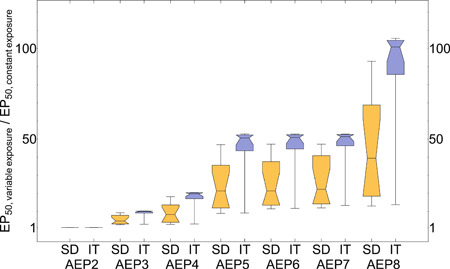
Box‐Whisker plots, showing distributions of “normalized” EP50 values for aquatic exposure profiles (AEPs) 2 to 8, for the SD model (orange) and the IT model (blue). The notches show median values, upper and lower fences indicate minimum and maximum values, and upper and lower quartile ranges are indicated by the boxes for each 13 species. The figure shows EP50 values for time‐variable scenarios divided by the values for constant exposure (AEP1). This means, the values on the *y*‐axis quantify the reduction of risk for the time‐variable exposure profile in relation to constant exposure. Distributions of values per AEP show variability of species sensitivities. IT = individual tolerance; SD = stochastic death.

The selected theoretical exposure profiles AEP1 to AEP8 were characterized by the same peak concentration (0.01 µg/L) but decreasing overall exposure when going from AEP1 to AEP8 (Figure 1). Dividing the calculated EP50 values for the theoretical exposure profiles AEP2 to AEP8 by the calculated EP50 of AEP1, that is, the exposure profile with a constant long‐term exposure, gave insight in the environmental risks of time‐variable exposures being reduced relative to constant exposure (Figure 4). In general, the risk reduction was larger when overall exposure was lower. Furthermore, the predicted reduction in environmental risk due to CPF was overall larger when based on the GUTS‐RED‐IT than on GUTS‐RED‐SD models. Although AEP5 to AEP7 were characterized by 2 pulsed exposures with the same peak concentrations but different time intervals between pulses, nevertheless the differences in reduction of environmental risks between AEP5 to AEP7 appeared to be small. Differences in predicted risks between exposure profiles AEP5 and AEP7 varied much less for the GUTS‐RED‐IT model than for the GUTS‐RED‐SD model. Differences in EP50 values between AEP5 and AEP7 were less than 5% for 10 of 13 species when the GUTS‐RED‐IT model was used, whereas in the case of the GUTS‐RED‐SD model for 10 of 13 species, the EP50 values varied more than 5% between AEP5 and AEP7 (see Supplemental Data SI Table 9).

### Risk assessment based on standard test species approach (Tier‐1 and Tier‐2C_1_)

Tier‐1 is the most conservative approach because it considers experimental data obtained from tests performed under constant exposure. Of the 2 most sensitive Tier‐1 species selected for our case study (see Table 1), the crustacean *D. magna* (96‐h EC50 = 0.17 µg/L) had a similar but slightly higher sensitivity to CPF than did the insect *Chaoborus obscuripes* (96‐h EC50 = 0.18 µg/L). The Tier‐1 TER of 17 (0.17/0.01) was lower than 100 (the Tier‐1 AF for acute assessment), indicating high acute risks for any aquatic exposure profiles.

The Tier‐2C_1_ approach is based on a less conservative approach because it considers refined time‐variable AEPs. A risk assessment using GUTS‐RED‐SD models for CPF and the 2 “standard” test species *D. magna* and *Chaoborus obscuripes* revealed consistently lower EP50 values for *D. magna*. In addition, these EP50 values gradually increased when going from AEP1 (EP50 = 1) to AEP8 (EP50 = 47), indicating that risks became smaller if the overall exposure also decreased (Table 2 and Supplemental Data SI Table 2). Application of the GUTS‐RED‐IT models revealed that EP50 values for *Chaoborus obscuripes* were overall lower than those for *D. magna*. Again these EP50 values gradually increased when going from AEP1 (EP50 = 4) to AEP8 (EP50 = 322) (Table 2 and Supplemental Data SI Table 2). Overall, for the evaluated data set, the risks assessed from GUTS‐RED‐SD models were stricter (lower EP50 values) than the ones from GUTS‐RED‐IT models. In general, results of both GUTS‐RED models should be reported and the most conservative be further used for the risk assessment; this implies that both types of models have been properly calibrated and validated (EFSA PPR et al. 2018). In this example case, although validation was not performed, we assumed for illustrative purposes that both the GUTS‐RED‐SD and GUTS‐RED‐IT models passed the validation step for all relevant species; hence here the most conservative EP50 values should be used in the risk assessment. The EP50 values calculated with the GUTS‐RED‐SD model for *D. magna* revealed that risks could not be excluded for any of the evaluated exposure profiles by this Tier‐2C_1_ approach because the corresponding EP50 values were lower than 100 (the Tier‐1 AF).

**Table 2:** Comparison of the risk assessment based on GUTS‐RED models in Tier‐2C_1_ (based on models for standard test species) and Tier‐2C_2_ (based on models for standard and additional test species) and ERAs based on experimental data in provisional Tier‐1 (standard test species approach), Tier‐2A (geometric mean approach) and Tier‐2B (SSD approach)^a^

Tier‐2C_1_ based on GUTS‐RED models and lowest exposure profile‐specific EP50 value for the combination *Daphnia magna* and *Chaoborus obscuripes*									Tier‐1 TER based on lowest 96‐h EC50 of standard test species selected and PEC_max_		
GUTS‐RED model	AEP1	AEP2	AEP3	AEP4	AEP5	AEP6	AEP7	AEP8	AEP1–AEP8	Low risk	Detailed information
SD	1^b^	1^b^	5^b^	9^b^	24^b^	25^b^	27^b^	47^b^	17^b^ (based on EC50 of *D. magna*)	Value >100	Supplemental Data SI Table 2
IT	4^b^	4^b^	33^b^	63^b^	166^c^	166^c^	166^c^	322^c^			

Tier‐2C_2_ based on GUTS‐RED models for the crustaceans *Asellus aquaticus, D. magna*, and *Gammarus pulex* and the insects *Chaoborus obscuripes, Cloeon dipterum*, and *Plea minutissima* and exposure profile‐specific lowest geometric mean EP50 for crustaceans and insects									Tier‐2A TER based on lowest geometric mean 96‐h EC50 values for the 3 crustaceans and 3 insects selected and PEC_max_		
GUTS‐RED model	AEP1	AEP2	AEP3	AEP4	AEP5	AEP6	AEP7	AEP8	AEP1–AEP8		
SD	5^b^	5^b^	29^b^	49^b^	127^c^	138^c^	149^c^	250^c^	42^b^ (based on geometric mean EC50 of insects)	Value >100	Supplemental Data SI Table 3 Supplemental Data SI Table 4
IT	7^b^	8^b^	69^b^	132^c^	339^c^	341^c^	347^c^	665^c^			

Tier‐2C_2_ based on GUTS‐RED models for all 13 arthropod taxa and exposure profile‐specific HP5 values derived from SSDs									Tier‐2B TER based on the HC5 from an SSD constructed with 96‐h EC50 values of all 13 arthropods and PEC_max_		
GUTS‐RED model	AEP1	AEP2	AEP3	AEP4	AEP5	AEP6	AEP7	AEP8	AEP3 – AEP8		
SD	1.0^b^	1.4^b^	7.2^c^	11.0^c^	30.0^c^	33.0^c^	35.0^c^	51.0^c^	7.9^c^ (according to EFSA PPR 2013, valid only in case of pulsed exposures, so AEP3 to AEP8)	Value >3–6	Supplemental Data SI Table 5 Supplemental Data SI Table 6
IT	1.6^b^	1.9^b^	14.0^c^	23.0^c^	60.0^c^	65.0^c^	67.0^c^	120^c^			

AEP = aquatic exposure profile (see Figure 1); AF = assessment factor; EFSA = European Food Safety Authority; ERA = environmental risk assessment; GUTS‐RED = reduced General Unified Threshold models of Survival; IT = individual tolerance; PEC_max_ = highest predicted exposure concentration; SD = stochastic death; SSD = species sensitivity distribution; TER = toxicity exposure ratio.

^a^ To facilitate comparisons, the provisional Tier‐1, Tier‐2A, and Tier‐2B assessments are expressed in terms of TERs.

^b^ EP50 values and TERs smaller than the related assessment factor, indicating potential high risk.

^c^ Values larger than the AF, indicating low risk.

### Risk assessment based on the geometric mean approach (Tier‐2A and Tier‐2C_2_ geomean)

As for Tier‐1, the Tier‐2A approach considers experimental data obtained from tests performed under standard or constant exposure but on additional species. Based on the toxicity data mentioned in Table 1, the geometric mean 96‐h EC50 for the 3 selected aquatic crustaceans *Asellus aquaticus* (3.43 µg/L), *D. magna* (0.17 µg/L), and *G. pulex* (0.23 µg/L) was 0.51 µg/L. For the 3 selected insects, *Chaoborus obscuripes* (0.18 µg/L), *Cloeon dipterum* (0.31 µg/L), and *Plea minutissima* (1.29 µg/L), the geometric mean 96‐h EC50 was 0.42 µg/L. In the Tier‐2A approach, the lowest value of the geometric means calculated for crustaceans and insects respectively was used as the effect estimate in the risk assessment, namely 0.42 µg/L. Using the PEC_max_ of 0.01 µg/L for all evaluated exposure profiles, the Tier‐2A TER became 0.42/0.01 = 42, which was <100, thus indicating their potential high acute risk.

The Tier‐2C_2_ geomean approach also focuses on additional species but considers in addition refined aquatic exposure profiles; it is thus a less conservative approach than Tiers 2A or 2B, both based on standard exposure conditions. A risk assessment using GUTS‐RED‐SD models for CPF and 6 arthropods (Tier‐2C_2_) revealed that the geometric mean EP50 values for all the exposure profiles (AEP1–AEP8) were consistently lower for the 3 crustaceans (range 5–250) than for the 3 insects (range 20–510) (Table 2 and Supplemental Data SI Table 3). Although EP50 values were estimated with their uncertainty limits, uncertainties have been ignored when calculating the geometric mean. Because the geometric mean EP50 values calculated for crustaceans were higher than 100 (the Tier‐1 AF) for exposure profiles AEP5 to AEP8, the environmental risk for these exposure profiles were considered to be low (Figure 2). The ERA based on the geometric mean approach and GUTS‐RED‐SD models (Supplemental Data SI Table 3) was again stricter than the one using GUTS‐RED‐IT models (Supplemental Data SI Table 4), which indicated again potentially high acute risks for AEP1 to AEP4 (Table 2 and Figure 2). The modeling results revealed that the Tier‐2C_2_ risk assessment, based on GUTS‐RED models and the geometric mean approach, was consistent with our a priori assumption that geometric mean EP50 values would increase when going from AEP1 to AEP8. Notably, as was observed already in Tier‐2C_1_, also in Tier‐2C_2_ the geometric mean EP50 values calculated with GUTS‐RED‐IT models were lower for insects than those for crustaceans for nearly all exposure profiles; this is in contrast to the results of the GUTS‐RED‐SD models for which crustaceans consistently had lower EP50 values for all the exposure profiles

### Risk assessment based on the species sensitivity distribution approach (Tier‐2B and Tier‐2C_2_ SSD)

**Figure 5:**
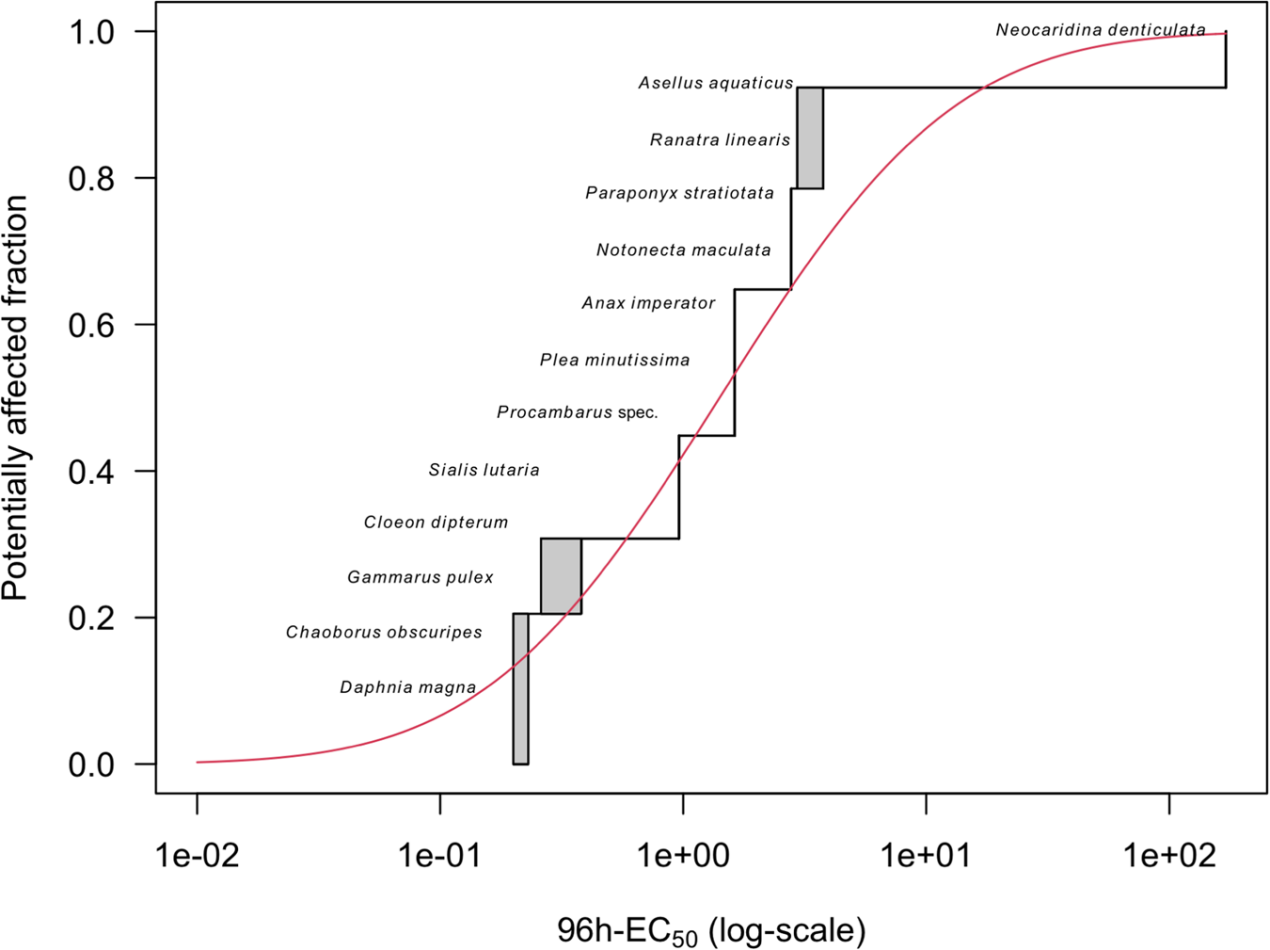
Species sensitivity distribution graph constructed with 96‐h EC50 values for the 13 arthropods presented in Table 1, and calculated HC5 values using MOSAIC_SSD_. MOSAIC_SSD_ = statistical model for species sensitivity distributions (Charles et al. 2018).

As for Tier‐2A, the Tier‐2B approach also considers experimental data obtained from tests performed under standard or constant exposure on additional species, but now with more species. For the Tier‐2B SSD approach, the 96‐h EC50 estimates for all species provided in Table 1 were used as input for MOSAIC_SSD_. The constructed SSD is presented in Figure 5 and the estimated median HC5 value was 0.079 µg/L. According to the EFSA AGD (EFSA PPR 2013), an AF of 3 to 6 has to be applied to this median HC5. Using the PEC_max_ of 0.01 µg/L would result in a Tier‐2B TER of 7.9 (0.079/0.01), which was above the suggested AF range of 3 to 6 used in the acute Tier‐2B approach for invertebrates, so risks for pulsed exposure profiles AEP3 to AEP8 were identified as low. Note that EFSA PPR (2013) states that only acute SSDs can be used in the acute risk assessment for pulsed exposure regimes, thus excluding AEP1 and AEP2 from our analysis (Figure 1).

A Tier‐2C_2_ SSD risk assessment using both GUTS‐RED models for CPF and the 13 arthropods resulted again in exposure profile‐specific HP5 values consistently lower for the GUTS‐RED‐SD models than for the GUTS‐RED‐IT models (Table 2; Supplemental Data SI Tables 5 and 6). Nevertheless, both model types indicated that potential risks were high for AEP1 and AEP2, given that in these 2 cases the exposure profile‐specific median HP5 values were smaller than 3 to 6, whereas for AEP3 to AEP8 risks were identified to be low, based on median HP5 values higher than 3 to 6 (see Figure 2). Furthermore, the exposure‐profile specific median HP5 values were consistent with our a priori assumption that they would increase when going from AEP1 to AEP8.

### Comparison of Tier‐2C and Tier‐3 risk assessment

In general, according to the principles of the tiered approach, a Tier‐2C risk assessment should not be less conservative than a Tier‐3 risk assessment. However, this principle should hold true only when considering all relevant effects over the relevant exposure duration. The comparison performed in the present manuscript is between a Tier‐2C based on acute laboratory tests, where only mortality was assessed, and an experimental ditch study, where potentially long‐term sublethal effects may take place. There are cases in which the main effects at population levels are not due to induced mortality: in those cases, the outcome of a Tier‐3 experiment might be more conservative than a Tier‐2C based on acute data. Nevertheless, for CPF, the main effect is mortality and the exposure in the Tier‐3 experiment is rather short. Hence, the expectation is that the Tier‐2C will be more conservative than the Tier‐3.

The 0.1 µg a.s./L treatment level from the experimental ditch study (see Figure 3) that resulted in an Effect class 2 concentration for *G. pulex* only, could be used to derive a provisional Tier‐3 ETO‐RAC by applying an AF of 2 to 3 (= 0.033 – 0.05 µg a.s./L). This implies that when dividing the measured concentration values of the 0.1 µg a.s./L treatment by 2 or 3 (according to the AFs), the resulting exposure profiles should not indicate high risks if evaluated with the available GUTS‐RED models for the 13 aquatic arthropods and by using the SSD approach as done in Tier‐2C_2_. Using the measured experimental ditch exposure profiles as input for the GUTS‐RED models available for the 13 aquatic arthropods, EP50 values for each compound–species combination and each reduced experimental ditch exposure profile were calculated, as well as the corresponding HP5 on basis of the SSD approach (Supplemental Data SI Table 7). Using the exposure profile of the 0.1 µg a.s./L treatment level that corresponds to the NOEC divided by a factor of 2, the calculated median HP5 and their corresponding 95% confidence intervals were 1.8 (0.54–9.9) and 2.8 (1.2–11) for both the GUTS‐RED‐SD and the GUTS‐RED‐IT models, respectively. When divided by a factor of 3, the resulting HP5 values were 2.55 (0.65–14) and 4.4 (1.8–17) for the GUTS‐RED‐SD and the GUTS‐RED‐IT models, respectively (Supplemental Data SI Table 7). The median HP5 values on the basis of the GUTS‐RED‐SD models were below the range of 3 to 6 (AFs for the SSD approach based on GUTS models; Figure 2), so that high acute risks could not be excluded. These results showed that the Tier‐2C_2_ risk assessment based on the SSD approach indeed was more conservative than the Tier‐3 approach, when evaluating the (reduced) exposure profile of the experimental ditch study that is in line with the Tier‐3 ETO‐RAC. Further verification of the overall GUTS approach was provided by checking predicted survival by the calibrated GUTS‐RED‐SD and GUTS‐RED‐IT models against the relative abundances in the mesocosm after 4 wk for *Cloeon dipterum, Chaoborus obscuripes, G. pulex*, and *Asellus aquaticus* (Supplemental Data SI Table 8). The GUTS modeling does not account for population dynamics, but the comparison between predicted survival and abundance of these 4 species in the mesocosm revealed that in most cases GUTS predictions accurately matched the observed relative abundance of the 4 selected species in the mesocosm tests. This accurate prediction is most likely related to the insecticidal mode of action of the test compound and its relatively short time to onset of effects on mortality and immobility. For a substance that needs a longer time to onset of (sublethal) effects, the outcome of a risk assessment based on Tier‐2C GUTS and Tier‐3 tests would be expected to be more different.

## DISCUSSION

### Possibilities and boundaries for GUTS applications in risk assessment

The GUTS models provide more possibilities to aquatic risk assessment than to allow for only a specific refinement option. It can, for example, be used in Tier‐1 to analyze data from standard acute tests, in Tier‐2 to predict survival under untested conditions (e.g., with time‐varying exposure as presented in the present paper), or in Tier‐3 as a module for modeling toxic lethal effects in population models. In addition, GUTS models can be used as a research tool to address relevant regulatory aspects, for example, to evaluate the possible toxicological (in)dependence of different pulses, to select the most relevant time frame of an annual exposure profile for higher tier effect assessment, to design refined exposure experiments that can be used to validate a GUTS model, to explore reciprocity of effects, or to identify problematic compounds that show slow or fast uptake kinetics or possible irreversible binding of the chemical to the target site.

The present paper illustrated the possible implementation of GUTS models in the risk assessment for addressing a specific type of refinement, namely the Tier‐2C approach. This approach was proposed in the EFSA AGD as an option for predicting lethal effects under time variable exposure (EFSA PPR 2013). Until now, the Tier‐2C approach was usually applied using experimental data, but the present paper shows an example of how it might be used based on GUTS modeling. It should be noted that, independently of using experiments or models, this refinement option includes some critical points and open issues, for example, about the co‐occurrence of the most sensitive life stage of the organisms and peak concentrations. The level of protection reached with a Tier‐2C approach in regulatory risk assessment is therefore under discussion among the European Member States authorities. As recently reported (EFSA et al. 2019), concerns related to using Tier‐2C as a refinement option will be addressed in the context of a future revision of the AGD (EFSA PPR 2013), but were not analyzed in the present paper. Therefore, the present paper does not aim to promote the Tier‐2C approach nor to critically assess it, but evaluates instead the possibility of GUTS modeling as a relevant tool in regulatory risk assessment.

### Model validation

According to EFSA PPR et al. (2018), the regulatory use of GUTS models for risk assessment requires that these models are validated by means of independent laboratory experiments characterized by time‐variable exposure. The minimum requirements for validation experiments are the testing of 2 exposure profiles with at least 2 pulses each; for each pulse, at least 3 concentrations should be tested, leading to low, medium, and strong effects. Basically, using the GUTS modeling approach in a regulatory context as we did in the present paper for illustrative purposes would have required to have validated the GUTS‐RED models first for each selected species (in this example, 13 arthropods) with suitable validation data in order to establish that they were fit‐for‐purpose as tools in the Tier‐2C risk assessment of pesticides. Independent validation experiments were performed with CPF and *Asellus aquaticus* and *N. denticulata* (Rubach 2010) and *D. magna, Chaoborus obscuripes, Cloeon dipterum*, and *Plea minutissima (*Zafar 2012), but GUTS‐RED models were not yet systematically validated using at least these experimental results for CPF. For the modeling work presented in the present paper, we thus assumed that the GUTS‐RED models performed well in predicting the effects of time‐variable exposures for all the 13 species, without demonstrating this explicitly. A study on the calibration and validation of GUTS‐RED models for a set of aquatic invertebrates and neonicotinoid compounds gives an example on how such a model validation study can be performed based on additional laboratory experiments (Focks et al. 2018). To validate the protectiveness of the risk assessment procedure based on GUTS models, results of a valid semifield test may be used, as was done, for example, in the present paper by evaluating the exposure profile of a mesocosm study from which the Tier‐3 ETO‐RAC is derived.

### Use of GUTS models within the tiered decision schemes of the EFSA aquatic guidance document

The present investigation with the insecticide CPF indicates that the prediction of the effects from different time‐variable exposures on survival or immobility of species depends on 1) the type of GUTS‐RED model used, namely SD versus IT; 2) the species selected; and 3) the type of exposure profile. With respect to application in a regulatory context, EFSA PPR et al. (2018) recommended using both GUTS‐RED‐SD and GUTS‐RED‐IT models because currently it cannot be anticipated which model type will deliver the most conservative outputs (Nyman et al. 2012; Ashauer et al. 2016; Baudrot, Preux et al. 2018; Focks et al. 2018). Then, based on both model outputs and given that both models have proven sufficient prediction quality in validation tests, the most conservative predictions should be selected for the Tier‐2C risk assessment. On the basis of data for neonicotinoid insecticides and several aquatic arthropod species (comprising crustaceans and insects), Focks et al. (2018) also concluded that the application of GUTS modeling for refinements in ERA appeared possible for most of the presented species–compound combinations, when the results of both the GUTS‐RED‐SD and GUTS‐RED‐IT models were used. Gabsi et al. (2018) advocated that the selection of the model type (SD or IT) to be used in the risk assessment should be based on those toxicodynamic parameters that best reflect the toxic mode of action of the compound under evaluation. For example, they showed that the GUTS‐RED‐IT model parameters more successfully captured the slow‐acting mechanism of the herbicide tembotrione when predicting the response of time‐variable exposures on the mysid shrimp *Americamysis bahia* than the GUTS‐RED‐SD.

To get more mechanistic insight into the predictive value of the GUTS‐RED‐SD and GUTS‐RED‐IT models, comparative studies on more species–compound combinations are required for taxa that differ in their biological traits and for compounds that differ in their toxic mode of action. For the time being, evaluating environmental risk of time‐variable exposures of pesticides on nontarget aquatic organisms in a regulatory context can best be done by applying both GUTS‐RED model types (SD and IT) and by selecting the most conservative predictions, unless it is clearly demonstrated that one of these models better captures the mechanism of action of the toxicant and the pattern of reduction in survival over time for the specific species used in the Tier‐2C risk assessment. In this context, the calibration and validation laboratory experiments required for each compound–species combination may have shed light on this.

### Understanding overall exposure patterns

The predicted reduction in environmental risks due to CPF was overall larger when based on the GUTS‐RED‐IT than on GUTS‐RED‐SD models. This appears to be consistent with known characteristics of the GUTS‐RED‐IT model, which uses the maximum internal damage over time for the effect modeling. Consequently, the time interval between exposure peaks was expected to be of less importance for the GUTS‐RED‐IT than for the GUTS‐RED‐SD model, given that the latter simulates hazard dynamics as a dynamical process. Any attempt to correlate model parameters with the differences in EP50 values between AEP5 and AEP7 resulted in low evidence for this (see Supplemental Data SI Table 9). The reason might be that GUTS‐RED models use a minimal parameter set. The dominant rate constant of a GUTS‐RED model is a “lumped” parameter, which is supposed to incorporate the most dominant processes of elimination and damage recovery, but at the same time also determines the dynamics of the internal damage recovery (Jager and Ashauer 2018). Some species showed no or low sensitivity against the time interval between the peaks, for example, *Chaoborus obscuripes* and *G. pulex*, which indicated that either the repair from damage needed less than 2 d or more than 10 d (the time interval between the pulses in AEP7). Other species, for example, *Anax imperator* or *Paraponyx stratiotata*, showed significant differences of up to 49% for the different no‐exposure intervals, which indicated that for some species the time interval between the pulses can play an important role. The median EP50 values, however, did not differ very much for the different time intervals between peak concentrations in AEP5 to AEP7. Consequently, the time interval between the pulses appeared of limited importance in SSD‐based evaluations of the EP50 values in the case of CPF.

### Consistency of the tiered approach when GUTS‐RED models are used as ERA tools

The example data set for the insecticide CPF and the assessments presented in the *Results* section illustrate that the proposals by EFSA PPR et al. (2018) on the use of GUTS‐RED models in Tier‐2C_1_ and Tier‐2C_2_ risk assessments (see Figure 2) are not in conflict with the experimental procedures described in the EFSA AGD (EFSA PPR 2013) and with the principle of the tiered approach, that is, lower tiers should be more conservative than higher ones. The risk assessments based on GUTS‐RED models presented in the present paper illustrate that the assessments become less conservative when going from Tier‐2C_1_ (for 2 standard test arthropod species) to geometric mean Tier‐2C_2_ (geometric mean approach for 2 standard and 4 additional arthropods) to SSD Tier‐2C_2_ (SSD approach for 13 arthropods) (see Table 2). Furthermore, the Tier‐2C_2_ assessment based on GUTS‐RED models for 13 arthropods resulted in a more conservative risk assessment than in a Tier‐3 (semifield study) assessment when using the CPF exposure profile of the semifield study that could be directly linked to the provisional Tier‐3 ETO‐RAC (indicative for the threshold level of effects). In mesocosm experiments, not only water exposure but also sediment and food exposure may play a role in the treatment‐related responses observed. Note, however, that CPF is characterized by a relatively short time to onset of lethal effects. Water exposure directly after CPF application and mortality or immobility effects on arthropods dwelling in the water column are captured well in the mesocosm experiment and most likely cover mortality effects on sediment‐dwelling organisms and exposure to pore water or sediment particles. Nevertheless, further exploration of the use of GUTS models in sediment ERA is an important topic for future research. In order to evaluate the consistency of the tiered approach when using GUTS‐RED models in the regulatory environmental risk assessment for pesticides, it is thus now recommended that similar studies be conducted with a representative number of substances differing in exposure dynamics and toxic mode of action. Further studies should also consider realistic AEPs, for example, predicted for different FOrum for Co‐ordination of pesticide fate models and their Use (FOCUS) surface‐water scenarios (FOCUS 2001).

## Disclaimer

The authors declare no conflicts of interest. The views expressed in this publication are those from the authors and do not necessarily represent the official position of EFSA.

## SUPPLEMENTAL DATA

Files contain 1) additional figures and tables with results and 2) raw data on survival and mobility as used to calibrate the GUTS models used within this article (the data have been documented in the publication Rubach et al. 2011).

**SI Table 1**. Parameter values and confidence intervals for the GUTS‐RED‐SD and the GUTS‐RED‐IT models

**SI Table 2**. EP50 values (and 95% confidence limits) calculated with GUTS‐RED‐SD and GUTS‐RED‐IT models for the 2 Tier‐1 test species (endpoint immobility) and the 8 constructed aquatic exposure profiles for chlorpyrifos (AEP1–AEP8)

**SI Table 3**. Tier‐2C_2_ geometric mean approach based on EP50 values (and 95% confidence limits) calculated with GUTS‐RED‐SD models for 3 aquatic crustaceans and 3 aquatic insects (endpoint immobility) and the 8 constructed aquatic exposure profiles for chlorpyrifos (AEP1–AEP8)

**SI Table 4**. EP50 values (and 95% confidence limits) calculated with GUTS‐RED‐IT models for 3 aquatic crustaceans and 3 aquatic insects (endpoint immobility) and the 8 constructed aquatic exposure profiles for chlorpyrifos (AEP1–AEP8)

**SI Table 5**. EP50 values (and 95% confidence limits) calculated with GUTS‐RED‐SD models for 13 aquatic arthropod taxa (endpoint immobility) and the 8 constructed aquatic exposure profiles for chlorpyrifos (AEP1–AEP8)

**SI Table 6**. EP50 values (and 95% confidence limits) calculated with GUTS‐RED‐IT models for 13 aquatic arthropod taxa (endpoint immobility) and the 8 constructed aquatic exposure profiles for chlorpyrifos (AEP1–AEP8)

**SI Table 7**. EP50 values (and 95% confidence limits) calculated with GUTS‐RED‐SD and GUTS‐RED‐IT models for 13 aquatic arthropod taxa (endpoint immobility) and the reduced (by a factor of 2 or 3) measured exposure profiles at 0.1 µg a.s./L treatment levels corresponding to the NOEC for chlorpyrifos in the experimental ditch study

**SI Table 8**. GUTS model predictions of survival in mesocosm tests. Measured exposure profiles in the mesocosm (see Figure 3) were used as input for the reduced GUTS SD and IT models for 4 species: *Cloeon dipterum, Chaoborus obscuripes, Gammarus pulex*, and *Asellus aquaticus*

**SI Table 9**. EP50 values for AEP5–AEP7, and relative changes

**Figure SI‐1**. Part 1 of calibration plots of the GUTS‐RED‐SD and the GUTS‐RED‐IT models.

**Figure SI‐2**. Part 2 of calibration plots for the GUTS‐RED‐SD and the GUTS‐RED‐IT models.

## Supporting information

This article contains online‐only Supplemental Data.

Supporting information.

Supporting information.

## Data Availability

All underlying data are published or made available in the Supplemental Data.
