# Supplementary material for: Application of General Unified Threshold Models of Survival Models for Regulatory Aquatic Pesticide Risk Assessment Illustrated with an Example for the Insecticide Chlorpyrifos
Source: Integr Environ Assess Manag. 2020 Sep 24;17(1):243–58. doi: 10.1002/ieam.4327 (PMC7821141; doi:10.1002/ieam.4327)
Supplement: Supplementary file 1 — Supporting information. [file IEAM-17-243-s001.docx]

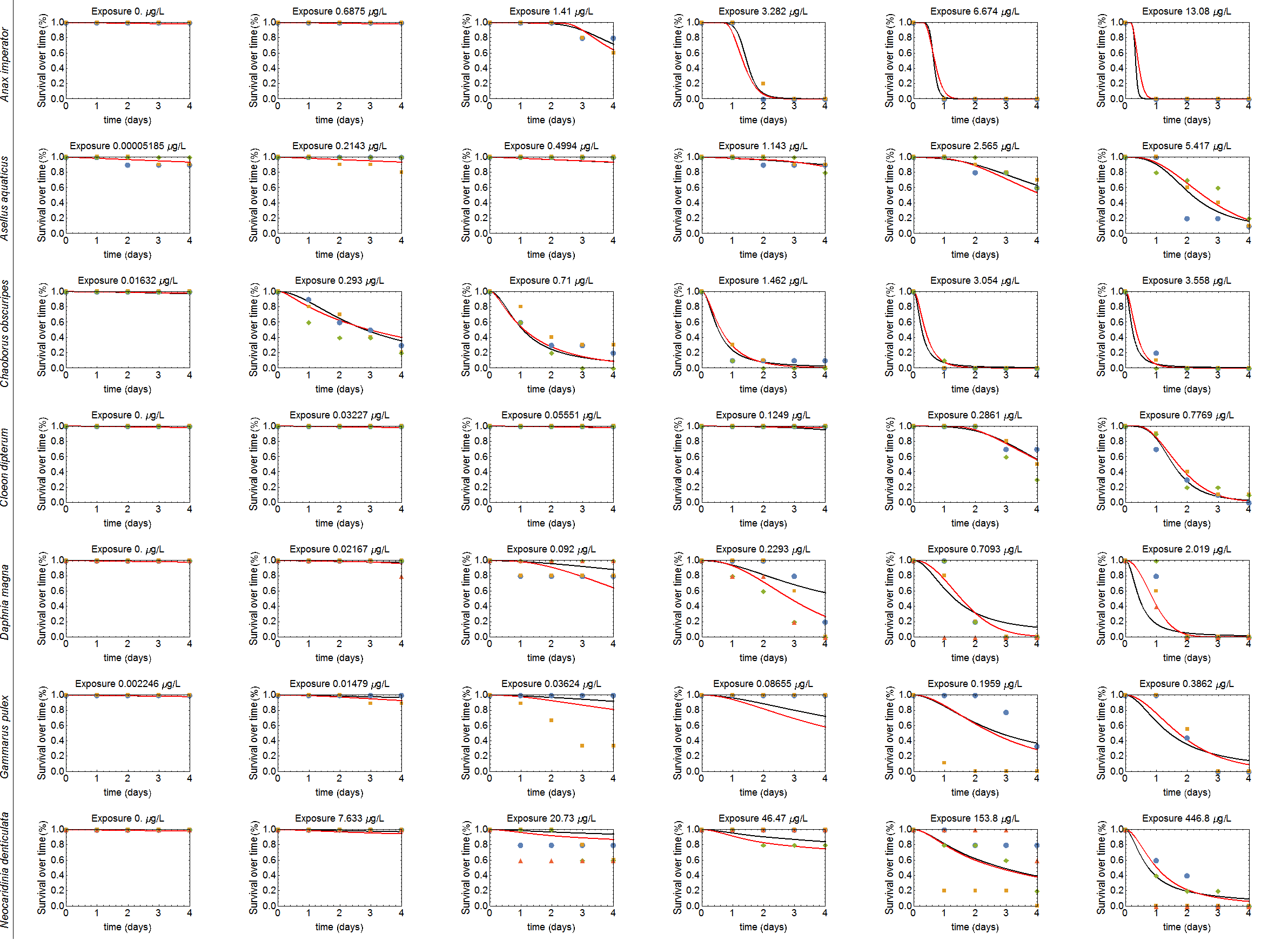


**Figure SI-1**: Part 1 of Calibration plots of the GUTS-RED-SD and the GUTS-RED-IT models. Black solid lines show the calibrated GUTS-RED-SD model, red lines the GUTS-RED-IT model. Symbols depict the observed survival rates. Numbers of survivors have been transformed into relative abundances for the plots. Exposure levels for the single treatments are given in the respective plot titles. Species names are given on the left hand side.


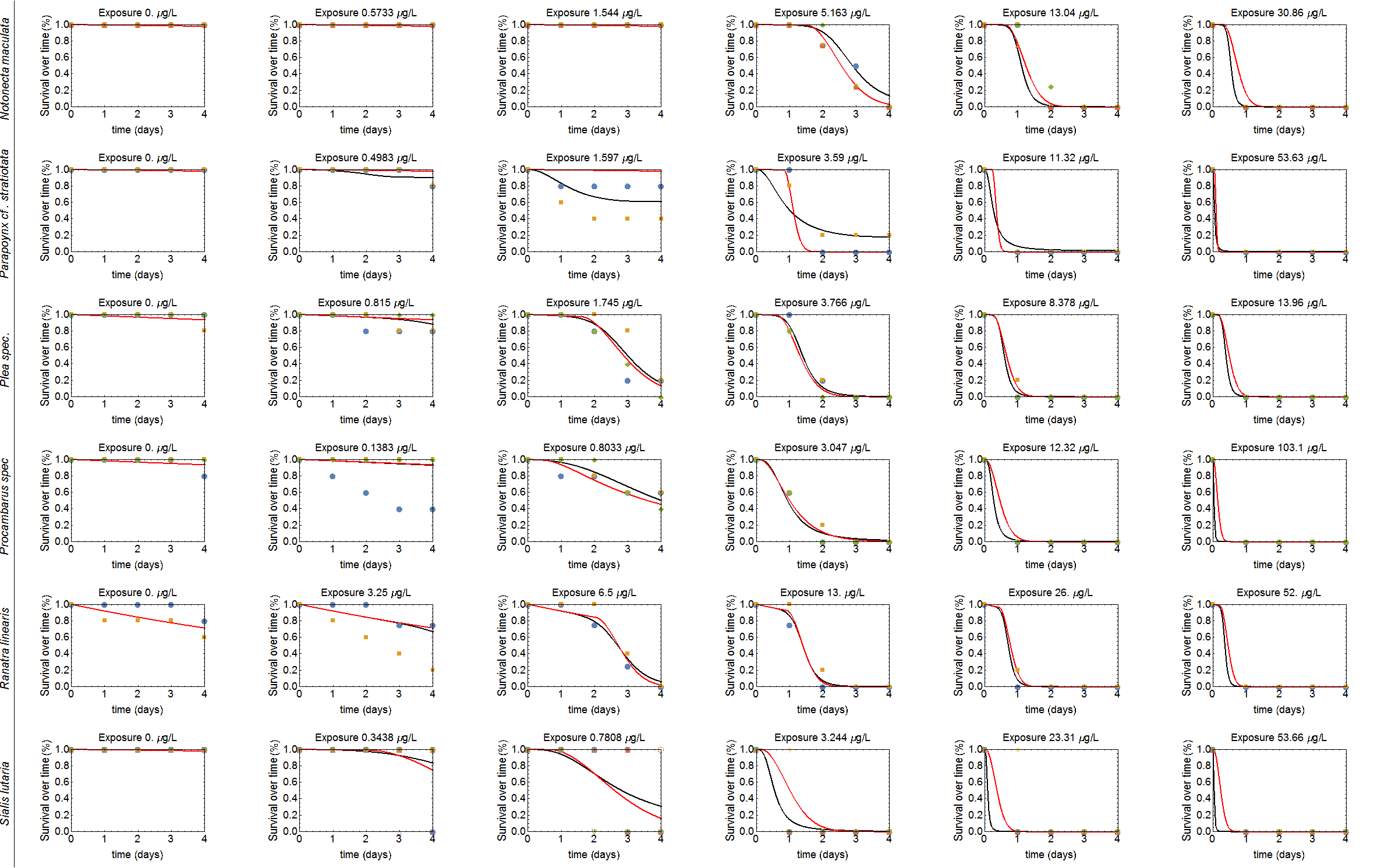


**Figure SI-2**: Part 2 of calibration plots for the GUTS-RED-SD and the GUTS-RED-IT models. Black solid lines show the calibrated GUTS-RED-SD model, red lines the GUTS-RED-IT model. Symbols depict the observed survival rates. Numbers of survivors have been transformed into relative abundances for the plots. Exposure levels for the single treatments are given in the respective plot titles. Species names are given on the left hand side.

**SI table 1**: Parameter values and confidence intervals (in brackets) for the GUTS-RED-SD and the GUTS-RED-IT models. If numerical approximation of confidence limits was not converging within 2 orders of magnitude above/below the respective optimal parameter value, the approximation routine was stopped and a hyphen (“-“) is shown.

|  | **GUTS-RED-SD** | | | | **GUTS-RED-IT** | | | |  |
| --- | --- | --- | --- | --- | --- | --- | --- | --- | --- |
| *Species* | **- LL-SD** | **kD** | **b** | **zw** | **- LL-IT** | **kD** | **mw** | **beta** | **hb** |
| *Anax imperator* | 15.523 | 0.514  (0.2523,1.011) | 3.446  (1.229,7.008) | 1.1  (0.7254,1.323) | 13.391 | 0.1814  (0.02349,0.3909) | 0.8151  (0.763,0.7904) | 9.367  (5.836,15.29) | 5.00E-03 |
| *Parapoynx cf. stratiotata* | 27.646 | 0.4129  (0.235,0.5748) | 14.06  (3.447,134.2) | 1.286  (0.9192,1.483) | 22.076 | 0.3549  (0.2898,0.937) | 1.339  (1.339,1.611) | 2.322  (1.374,3.645) | 5.00E-03 |
| *Notonecta maculata* | 28.849 | 0.03815  (-,0.2209) | 6.744  (1.155,19.85) | 0.3453  (0.08021,1.558) | 32.586 | 3.7E-3  (-,0.04712) | 0.0599  (0.0599,0.07111) | 7.836  (5.326,11.6) | 5.00E-03 |
| *Sialis lutaria* | 32.927 | 0.04158  (-,0.6299) | 10.49  (2.591,65.86) | 0.02705  (-,0.1303) | 33.679 | 4.7E-4  (1.5E-5,2.7E-3) | 1.1E-3  (5.6E-4,1.8E-3) | 3.179  (2.021,4.645) | 5.00E-03 |
| *Ranatra linearis* | 44.138 | 0.1965  (-,0.66) | 2.369  (0.5193,13.22) | 2.131  (-,4.497) | 44.010 | 5.3E-4  (4.3E-5,-) | 9.9E-3  (9.3E-3,0.01414) | 7.289  (4.271,14.17) | 0.08456 |
| *Plea spec.* | 49.731 | 0.4192  (0.1041,0.8448) | 2.472  (1.17,5.07) | 0.8475  (0.275,1.233) | 49.427 | 8.5E-3  (1.4E-3,0.2027) | 0.04457  (0.04216,0.06594) | 5.696  (4.078,7.969) | 0.01681 |
| *Procambarus spec* | 55.331 | 1.3  (0.4108,12.25) | 0.6035  (0.2286,1.549) | 0.3801  (0.2131,0.5532) | 54.667 | 0.03871  (-,0.311) | 0.1205  (0.1026,0.1809) | 3.03  (1.811,4.884) | 0.01681 |
| *Cloeon dipterum* | 76.645 | 0.4453  (0.1032,0.9632) | 3.397  (1.755,11.72) | 0.1318  (0.03062,0.2008) | 77.514 | 0.01106  (-,0.1941) | 0.01336  (0.0133,0.01789) | 3.802  (2.93,4.896) | 5.00E-03 |
| *Neocaridinia denticulata* | 78.948 | 2.254  (1.109,6.64) | 1.8E-3  (9.7E-4,2.6E-3) | 1.9  (-,72.23) | 77.286 | 1.7E-3  (-,0.08108) | 0.8036  (0.7051,1.233) | 1.755  (1.193,2.805) | 5.00E-03 |
| *Gammarus pulex* | 91.438 | 0.6942  (0.0565,2.731) | 2.345  (1.287,95.61) | 4.5E-3  (-,0.01816) | 97.082 | 1.1E-3  (-,-) | 6.3E-4  (5.5E-4,6.9E-4) | 1.882  (1.356,2.697) | 5.00E-03 |
| *Daphnia magna* | 103.058 | 0.03047  (-,0.3289) | 24.8  (2.767,530.2) | 1.5E-3  (-,0.02979) | 126.697 | 3.3E-5  (2.8E-6,1.7E-4) | 3.7E-5  (3.3E-5,3.6E-5) | 2.007  (1.552,2.62) | 5.00E-03 |
| *Asellus aquaticus* | 117.951 | 0.1695  (6.3E-3,0.9136) | 0.3389  (0.09426,11.47) | 0.3816  (0.02081,1.886) | 118.350 | 2.5E-3  (3.5E-4,0.1494) | 0.03335  (0.02964,0.05033) | 3.075  (2.294,4.069) | 0.01724 |
| *Chaoborus obscuripes* | 131.845 | 6.009  (1.962,84.39) | 0.9754  (0.7412,1.404) | 0.0498  (6.E-3,0.1243) | 130.424 | 7.5E-3  (7.3E-4,0.2127) | 6.4E-3  (6.1E-3,6.4E-3) | 1.96  (1.569,2.412) | 5.00E-03 |

**SI Table 2**: EP_50_ values (and 95% confidence limits) calculated with GUTS-RED-SD and GUTS-RED-IT models for the two Tier-1 test species (endpoint immobility) and the 8 constructed aquatic exposure profiles for chlorpyrifos (AEP1-AEP8). In this Tier-2C_1_ approach the EP_50_ should be > 100 (the AF in the acute Tier-1 effect assessment). EP_50_ values and TERs smaller than the related assessment factor indicating potential high risk are shown in red, values larger than the AF indicating low risk are shown in green.

| **GUTS-RED-SD** | **AEP1** | **AEP2** | **AEP3** | **AEP4** | **AEP5** | **AEP6** | **AEP7** | **AEP8** |
| --- | --- | --- | --- | --- | --- | --- | --- | --- |
| *Daphnia magna* | 1  (0-4) | 1  (1-5) | 5  (4-28) | 9  (6-42) | 24  (15-113) | 25  (16-126) | 27  (15-142) | 47  (32-211) |
| *Chaoborus obscuripes* | 7  (4-14) | 9  (4-17) | 35  (29-46) | 63  (53-83) | 161  (141-211) | 161  (131-211) | 161  (133-201) | 293  (247-385) |
| **Lowest EP_50_** | **1** | **1** | **5** | **9** | **24** | **25** | **27** | **47** |
|  |  |  |  |  |  |  |  |  |
| **GUTS-RED-IT** | **AEP1** | **AEP2** | **AEP3** | **AEP4** | **AEP5** | **AEP6** | **AEP7** | **AEP8** |
| *Daphnia magna* | 4  (3-5) | 5  (4-6) | 40  (33-47) | 81  (65-98) | 205  (173-256) | 205  (163-256) | 205  (167-244) | 410  (320-513) |
| *Chaoborus obscuripes* | 4  (3-4) | 4  (3-5) | 33  (27-41) | 63  (52-71) | 166  (125-187) | 166  (135-187) | 166  (135-208) | 322  (255-383) |
| **Lowest EP_50_** | **4** | **4** | **33** | **63** | **166** | **166** | **166** | **322** |

**SI Table 3**: Tier-2C2 geometric mean approach based on EP_50_ values (and 95% confidence limits) calculated with GUTS-RED-SD models for 3 aquatic crustaceans and 3 aquatic insects (endpoint immobility) and the 8 constructed aquatic exposure profiles for chlorpyrifos (AEP1-AEP8) . The EP_50_ values were used to calculate a geometric mean EP_50_ for each taxonomic group. In this approach the geometric mean EP_50_ should be > 100 (the AF in the acute Tier-1 effect assessment). EP_50_ values and TERs smaller than the related assessment factor indicating potential high risk are shown in red, values larger than the AF indicating low risk are shown in green.

|  | **AEP1** | **AEP2** | **AEP3** | **AEP4** | **AEP5** | **AEP6** | **AEP7** | **AEP8** |
| --- | --- | --- | --- | --- | --- | --- | --- | --- |
| Crustacea |  |  |  |  |  |  |  |  |
| *Asellus aquaticus* | 52  (15-188) | 59  (16-215) | 356  (103-590) | 527  (180-857) | 1406  (461-2373) | 1602  (500-2668) | 1797  (533-2695) | 2617  (920-4192) |
| *Daphnia magna* | 1  (0-4) | 1  (1-5) | 5  (4-28) | 9  (6-42) | 24  (15-113) | 25  (16-126) | 27  (15-142) | 47  (32-211) |
| *Gammarus pulex* | 2  (0-3) | 2  (0-3) | 13  (2-23) | 24  (5-43) | 63  (10-111) | 66  (14-115) | 68  (13-120) | 127  (15-214) |
| **Geometric mean EP_50_ Crustacea** | **5** | **5** | **29** | **49** | **127** | **138** | **149** | **250** |
| Insecta |  |  |  |  |  |  |  |  |
| *Chaoborus obscuripes* | 7  (4-14) | 9  (4-17) | 35  (29-46) | 63  (53-83) | 161  (141-211) | 161  (131-211) | 161  (133-201) | 293  (247-385) |
| *Cloeon dipterum* | 14  (5-20) | 16  (5-24) | 54  (35-59) | 67  (50-76) | 200  (138-230) | 239  (155-254) | 254  (179-266) | 322  (254-363) |
| *Plea minutissima* | 86  (38-126) | 97  (41-140) | 276  (192-303) | 305  (270-334) | 938  (729-1064) | 1182  (822-1255) | 1260  (994-1411) | 1406  (1252-1560) |
| **Geometric mean EP_50_ Insecta** | **20** | **24** | **81** | **109** | **311** | **357** | **372** | **510** |
|  |  |  |  |  |  |  |  |  |
| **Lowest Geometric mean EP_50_** | **5** | **5** | **29** | **49** | **127** | **138** | **149** | **250** |

**SI Table 4**: EP_50_ values (and 95% confidence limits) calculated with GUTS-RED-IT models for 3 aquatic crustaceans and 3 aquatic insects (endpoint immobility) and the 8 constructed aquatic exposure profiles for chlorpyrifos (AEP1-AEP8) . The EP_50_ values were used to calculate a geometric mean EP_50_ for each taxonomic group to explore the Geometric mean approach. In this approach the geometric mean EP_50_ should be > 100 (the AF in the acute Tier-1 effect assessment). EP_50_ values and TERs smaller than the related assessment factor indicating potential high risk are shown in red, values larger than the AF indicating low risk are shown in green.

|  | **AEP1** | **AEP2** | **AEP3** | **AEP4** | **AEP5** | **AEP6** | **AEP7** | **AEP8** |
| --- | --- | --- | --- | --- | --- | --- | --- | --- |
| Crustacea |  |  |  |  |  |  |  |  |
| *Asellus aquaticus* | 51  (43-69) | 59  (48-78) | 498  (421-607) | 977  (854-1160) | 2500  (2195-2969) | 2500  (2109-2969) | 2539  (2142-3223) | 5000  (4180-5625) |
| *Daphnia magna* | 4  (3-5) | 5  (4-6) | 40  (33-47) | 81  (65-98) | 205  (173-256) | 205  (163-256) | 205  (167-244) | 410  (320-513) |
| *Gammarus pulex* | 2  (2-6) | 3  (2-7) | 22  (18-32) | 44  (33-54) | 112  (88-140) | 112  (91-154) | 112  (86-158) | 225  (168-279) |
| **Geometric mean EP_50_ Crustacea** | **7** | **10** | **76** | **152** | **386** | **386** | **388** | **773** |
| Insecta |  |  |  |  |  |  |  |  |
| *Chaoborus obscuripes* | 4  (3-4) | 4  (3-5) | 33  (27-41) | 63  (52-71) | 166  (125-187) | 166  (135-187) | 166  (135-208) | 322  (255-383) |
| *Cloeon dipterum* | 5  (5-7) | 6  (6-9) | 48  (43-60) | 92  (81-103) | 234  (205-269) | 234  (211-278) | 244  (221-290) | 459  (402-527) |
| *Plea minutissima* | 22  (20-54) | 25  (22-91) | 205  (183-340) | 396  (346-445) | 1006  (887-1132) | 1016  (920-1325) | 1035  (938-1601) | 1992  (1805-2257) |
| **Geometric mean EP_50_ Insecta** | **8** | **8** | **69** | **132** | **339** | **341** | **347** | **665** |
|  |  |  |  |  |  |  |  |  |
| **Lowest Geometric mean EP_50_** | **7** | **8** | **69** | **132** | **339** | **341** | **347** | **665** |

**SI Table 5**: EP_50_ values (and 95% confidence limits) calculated with GUTS-RED-SD models for 13 aquatic arthropod taxa (endpoint immobility) and the 8 constructed aquatic exposure profiles for chlorpyrifos (AEP1-AEP8). The EP_50_ values were used to calculate Aquatic Exposure Profile-specific HP5 values (and 95% confidence intervals) using the MOSAIC SSD procedure. EP_50_ values and TERs smaller than the related assessment factor indicating potential high risk are shown in red, values larger than the AF indicating low risk are shown in green.

|  | **AEP1** | **AEP2** | **AEP3** | **AEP4** | **AEP5** | **AEP6** | **AEP7** | **AEP8** |
| --- | --- | --- | --- | --- | --- | --- | --- | --- |
| *Anax imperator* | 112  (71-134) | 123  (84-146) | 300  (269-311) | 322  (297-347) | 1016  (936-1047) | 1279  (1109-1289) | 1328  (1110-1484) | 1445  (1265-1626) |
| *Asellus aquaticus* | 52  (15-188) | 59  (16-215) | 356  (103-590) | 527  (180-857) | 1406  (461-2373) | 1602  (500-2668) | 1797  (533-2695) | 2617  (920-4192) |
| *Chaoborus obscuripes* | 7  (4-14) | 9  (4-17) | 35  (29-46) | 63  (53-83) | 161  (141-211) | 161  (131-211) | 161  (133-201) | 293  (247-385) |
| *Cloeon dipterum* | 14  (5-20) | 16  (5-24) | 54  (35-59) | 67  (50-76) | 200  (138-230) | 239  (155-254) | 254  (179-266) | 322  (254-363) |
| *Daphnia magna* | 1  (0-4) | 1  (1-5) | 5  (4-28) | 9  (6-42) | 24  (15-113) | 25  (16-126) | 27  (15-142) | 47  (32-211) |
| *Gammarus pulex* | 2  (0-3) | 2  (0-3) | 13  (2-23) | 24  (5-43) | 63  (10-111) | 66  (14-115) | 68  (13-120) | 127  (15-214) |
| *Neocaridinia denticulata* | 1563  (977-8270) | 1797  (1123-9308) | 15000  (10313-24375) | 28750  (19766-44922) | 75000  (51563-112500) | 75000  (51563-111328) | 75000  (51563-117188) | 150000  (103125-225000) |
| *Notonecta maculata* | 59  (38-163) | 68  (44-192) | 464  (319-774) | 801  (614-932) | 2090  (1534-2612) | 2207  (1612-3207) | 2402  (1802-3815) | 4023  (3097-4487) |
| *Paraponyx stratiotata* | 128  (93-167) | 143  (107-176) | 369  (352-380) | 383  (359-419) | 1162  (1143-1271) | 1514  (1437-1585) | 1660  (1517-1766) | 1729  (1634-1933) |
| *Plea minutissima* | 86  (38-126) | 97  (41-140) | 276  (192-303) | 305  (270-334) | 938  (729-1064) | 1182  (822-1255) | 1260  (994-1411) | 1406  (1252-1560) |
| *Procambarus spec.* | 42  (12-60) | 49  (14-67) | 127  (97-155) | 176  (143-242) | 527  (418-626) | 547  (444-686) | 547  (449-684) | 820  (679-1128) |
| *Ranatra linearis* | 219  (109-454) | 256  (144-499) | 986  (756-1144) | 1182  (979-1356) | 3262  (2701-3848) | 4023  (2969-4810) | 4883  (3588-5245) | 5625  (4659-6574) |
| *Sialis lutaria* | 5  (3-15) | 6  (4-17) | 44  (21-91) | 74  (40-119) | 195  (98-330) | 208  (130-389) | 227  (124-459) | 378  (189-591) |
|  |  |  |  |  |  |  |  |  |
| **HP_5_** | **1.0**  **(0.1-8.1)** | **1.4**  **(0.3-10)** | **7.2**  **(2.3-35.0)** | **11.0**  **(3.7-54.0)** | **30.0**  **(9.4-150)** | **33.0**  **(11.0-160)** | **35.0**  **(11.0-190)** | **51.0**  **(16.0-270)** |

**SI Table 6**: EP_50_ values (and 95% confidence limits) calculated with GUTS-RED-IT models for 13 aquatic arthropod taxa (endpoint immobility) and the 8 constructed aquatic exposure profiles for chlorpyrifos (AEP1-AEP8). The EP_50_ values were used to calculate Aquatic Exposure Profile-specific HP_5_ values (and 95% confidence intervals) using the MOSAIC SSD procedure. EP_50_ values and TERs smaller than the related assessment factor indicating potential high risk are shown in red, values larger than the AF indicating low risk are shown in green.

|  | **AEP1** | **AEP2** | **AEP3** | **AEP4** | **AEP5** | **AEP6** | **AEP7** | **AEP8** |
| --- | --- | --- | --- | --- | --- | --- | --- | --- |
| *Anax imperator* | 82  (29-123) | 96  (48-147) | 349  (256-399) | 425  (370-478) | 1143  (1031-1303) | 1387  (1170-1560) | 1670  (1250-1853) | 1953  (1709-2214) |
| *Asellus aquaticus* | 51  (43-69) | 59  (48-78) | 498  (421-607) | 977  (854-1160) | 2500  (2195-2969) | 2500  (2109-2969) | 2539  (2142-3223) | 5000  (4180-5625) |
| *Chaoborus obscuripes* | 4  (3-4) | 4  (3-5) | 33  (27-41) | 63  (52-71) | 166  (125-187) | 166  (135-187) | 166  (135-208) | 322  (255-383) |
| *Cloeon dipterum* | 5  (5-7) | 6  (6-9) | 48  (43-60) | 92  (81-103) | 234  (205-269) | 234  (211-278) | 244  (221-290) | 459  (402-527) |
| *Daphnia magna* | 4  (3-5) | 5  (4-6) | 40  (33-47) | 81  (65-98) | 205  (173-256) | 205  (163-256) | 205  (167-244) | 410  (320-513) |
| *Gammarus pulex* | 2  (2-6) | 3  (2-7) | 22  (18-32) | 44  (33-54) | 112  (88-140) | 112  (91-154) | 112  (86-158) | 225  (168-279) |
| *Neocaridinia denticulata* | 1719  (1396-3651) | 2031  (1627-3549) | 17500  (13021-24609) | 33750  (24785-46406) | 87500  (71094-120313) | 87500  (66992-123047) | 90000  (68906-123750) | 175000  (131250-240625) |
| *Notonecta maculata* | 63  (59-73) | 72  (68-84) | 605  (568-681) | 1201  (1107-1351) | 3066  (2851-3414) | 3086  (2869-3472) | 3125  (2930-3540) | 6094  (5673-6844) |
| *Paraponyx stratiotata* | 132  (115-262) | 146  (128-264) | 391  (311-537) | 410  (336-526) | 1211  (984-1637) | 1563  (1270-2100) | 1797  (1460-2253) | 1836  (1406-2295) |
| *Plea minutissima* | 22  (20-54) | 25  (22-91) | 205  (183-340) | 396  (346-445) | 1006  (887-1132) | 1016  (920-1325) | 1035  (938-1601) | 1992  (1805-2257) |
| *Procambarus spec* | 19  (15-28) | 22  (17-44) | 142  (115-194) | 244  (198-318) | 625  (508-820) | 664  (529-913) | 723  (587-994) | 1211  (946-1552) |
| *Ranatra linearis* | 70  (61-145) | 80  (67-172) | 693  (607-910) | 1377  (1162-1584) | 3555  (2994-4087) | 3555  (2999-4166) | 3555  (3111-4666) | 7109  (6166-8109) |
| *Sialis lutaria* | 9  (7-12) | 10  (8-13) | 88  (71-121) | 176  (132-222) | 459  (348-574) | 459  (351-574) | 459  (373-580) | 918  (688-1147) |
|  |  |  |  |  |  |  |  |  |
| **HP_5_** | **1.6**  **(0.6-5.9)** | **1.9**  **(0.8-7.9)** | **14.0**  **(5.7-51.0)** | **23.0**  **(9.9-86.0)** | **60.0**  **(26.0-240)** | **65.0**  **(28.0-240)** | **67.0**  **(28.0-260)** | **120**  **(50.0-420)** |

**SI Table 7**: EP_50_ values (and 95% confidence limits) calculated with GUTS-RED-SD and GUTS-RED-IT models for 13 aquatic arthropod taxa (endpoint immobility) and the reduced (by a factor of 2 or 3) measured exposure profiles at 0.1 µg a.s./L treatment levels corresponding to the NOEC for chlorpyrifos in the experimental ditch study. The EP_50_ values were used to calculate median HP_5_ values (and 95% confidence intervals) using the MOSAIC SSD procedure. EP_50_ values and TERs smaller than the related assessment factor indicating potential high risk are shown in red, values in the range of possible AF shown in yellow.

|  | **EP_50_ values corresponding to the measured exposure profile of 0.1 µg a.s./L treatment divided by a factor of 2** | | **EP_50_ values corresponding to the measured exposure profile of 0.1 µg a.s./L treatment divided by a factor of 3** | |
| --- | --- | --- | --- | --- |
|  | **GUTS-RED-SD** | **GUTS-RED-IT** | **GUTS-RED-SD** | **GUTS-RED-IT** |
| *Anax imperator* | 78.7  (70.1-83.7) | 84.8  (65.0-95.4) | 117  (107-127) | 127  (82.8-149) |
| *Asellus aquaticus* | 73.2  (19.5-17.4) | 92.8  (78.3-134) | 110  (29.2-264) | 139 (120-209) |
| *Chaoborus obscuripes* | 11.0  (7.6-15.5) | 6.7  (5.5-8.4) | 16.5  (10.3-22.5) | 10.1  (8.2-13.8) |
| *Cloeon dipterum* | 13,4  (7.2-17.4) | 10.1  (9.4-12.6) | 20.1  (10.8-26.8) | 15.3  (13.9-19.3) |
| *Daphnia magna* | 1.0  (0.7-5.8) | 7.3  (6.0-9.3) | 1.5  (0.9-8.8) | 11.0  (8.9-13.7) |
| *Gammarus pulex* | 2.9  (0.5-5.3) | 4.1  (3.3-8.0) | 4.3  (0.5-7.7) | 6.1  (5.0-11.8) |
| *Neocaridinia denticulata* | 2969  (1856-7857) | 3125  (2539-5859) | 4375  (3008-11758) | 4688  (3516-8345) |
| *Notonecta maculata* | 107  (65.5-179) | 116  (109-138) | 160  (96.1-27.1) | 175  (163-224) |
| *Paraponyx stratiotata* | 94.6  (93.0-104) | 97.7  (85.6-146) | 143  (141-153) | 146  (128-215) |
| *Plea minutissima* | 70.8  (46.7-84.1) | 41.5  (35.7-77.7) | 106  (71.3-124) | 62.3  (54.5-109) |
| *Procambarus spec* | 41.5  (23.3-52.3) | 34.2  (28.0-51.3) | 61.0  (33.6-75.5) | 51.3  (41.7-76.9) |
| *Ranatra linearis* | 231  (163-304) | 127  (111-234) | 349  (248-447) | 190  (167-333) |
| *Sialis lutaria* | 4.8  (3.1-10.4) | 8.1  (6.6-10.6) | 9.7  (4.3-21.5) | 16.2  (12.1-20.2) |
|  |  |  |  |  |
| **HP_5_** | **1.8**  (0.54-9.9) | **2.8**  (1.2-11) | **2.5**  (0.65-14) | **4.4**  (1.8-17) |

**SI table 8:** GUTS model predictions of survival in mesocosm tests. Measured exposure profiles in the mesocosm (see figure 3) were used as input for the reduced GUTS SD and IT models for 4 species: *C.dipterum*, *C.obscuripes*, *G.pulex* and *A.aquaticus*. Left column shows relative abundances in the mesocosm treatments after 4 weeks (Van Wijngaarden et al. 1996; Van den Brink et al. 1996).

| Nom. Conc. [µg/L] | Predicted surv. (SD) after 4 weeks | Predicted surv. (IT)  after 4 weeks | Abundance (% of control) at week 4 |
| --- | --- | --- | --- |
| *Cloeon dipterum* | | | |
| 44.0 | 0% | 0% | 0% |
| 6.0 | 0% | 0% | 0% |
| 0.9 | 25%^1^ | 14.7%^1^ | 12.3% |
| 0.1 | 100% | 100%^1^ | >100% |
| *Chaoborus obscuripes* | | | |
| 44.0 | 0% | 0% | 0% |
| 6.0 | 0% | 0.3% | 0% |
| 0.9 | 25.6% | 14.5% | 37% |
| 0.1 | 100% | 94.3% | 81.5% |
| *Gammarus pulex* | | | |
| 44.0 | 0% | 0% | 0% |
| 6.0 | 0% | 0.1% | 0% |
| 0.9 | 0.4% | 6.2% | 0% |
| 0.1 | 71.3% | 84.7% | 0%^1^ |
| *Asellus aquaticus* | | | |
| 44.0 | 0% | 0% | 0% |
| 6.0 | 7.1% | 29.6% | 14.6% |
| 0.9 | 100% | 99.5% | 0% |
| 0.1 | 100% | 100% | 19.5% |

^1^ Abundance was equal to control (100%) in week 2 and between 12-66% after week 4. It is unlikely that *Gammarus* has been wiped out from this treatment at any time point. All abundances were pretty low.

**SI table 9**: EP50 values for AEP5-7, and relative changes.

|  | **GUTS-RED-SD** | | | | **GUTS-RED-IT** | | | |
| --- | --- | --- | --- | --- | --- | --- | --- | --- |
|  | **EP50-AEP5** | **EP50-AEP6** | **EP50-AEP7** | **relative change AEP5-AEP7** | **EP50-AEP5** | **EP50-AEP6** | **EP50-AEP7** | **relative change AEP5-AEP7** |
| *Anax imperator* | 1016 | 1279 | 1328 | 30.8% | 1143 | 1387 | 1670 | 46.2% |
| *Asellus aquaticus* | 1406 | 1602 | 1797 | 27.8% | 2500 | 2500 | 2539 | 1.6% |
| *Chaoborus obscuripes* | 161 | 161 | 161 | 0.0% | 166 | 166 | 166 | 0.0% |
| *Cloeon dipterum* | 200 | 239 | 254 | 26.8% | 234 | 234 | 244 | 4.2% |
| *Daphnia magna* | 24 | 25 | 27 | 12.8% | 205 | 205 | 205 | 0.0% |
| *Gammarus pulex* | 63 | 66 | 68 | 7.7% | 112 | 112 | 112 | 0.0% |
| *Neocaridinia denticulata* | 75000 | 75000 | 75000 | 0.0% | 87500 | 87500 | 90000 | 2.9% |
| *Notonecta maculata* | 2090 | 2207 | 2402 | 15.0% | 3066 | 3086 | 3125 | 1.9% |
| *Parapoynx stratiotata* | 1162 | 1514 | 1660 | 42.9% | 1211 | 1563 | 1797 | 48.4% |
| *Plea spec.* | 938 | 1182 | 1260 | 34.4% | 1006 | 1016 | 1035 | 2.9% |
| *Procambarus spec* | 527 | 547 | 547 | 3.7% | 625 | 664 | 723 | 15.6% |
| *Ranatra linearis* | 3262 | 4023 | 4883 | 49.7% | 3555 | 3555 | 3555 | 0.0% |
| *Sialis lutaria* | 195 | 208 | 227 | 16.2% | 459 | 459 | 459 | 0.0% |
